# Use of High-Sensitivity Cardiac Troponin in Patients With Kidney Impairment: A Randomized Clinical Trial

**DOI:** 10.1001/jamainternmed.2021.1184

**Published:** 2021-06-07

**Authors:** Peter J. Gallacher, Eve Miller-Hodges, Anoop S.V. Shah, Atul Anand, Neeraj Dhaun, Nicholas L. Mills

**Affiliations:** 1 BHF Centre for Cardiovascular Science, University of Edinburgh, Edinburgh, Scotland; 2 Department of Renal Medicine, Royal Infirmary of Edinburgh, Edinburgh, Scotland; 3 London School of Hygiene and Tropical Medicine, London, England; 4 Usher Institute, University of Edinburgh, Edinburgh, Scotland

## Abstract

This randomized clinical trial examines the use of high-sensitivity cardiac troponin testing for patients with kidney impairment.

High-sensitivity cardiac troponin (hs-cTn) assays have improved the diagnosis of myocardial infarction in patients with healthy kidney function and are now widely used in clinical practice.^1^ However, in patients with kidney impairment, long-term elevations in troponin levels are common, and interpretation can be more challenging.^2^ As such, the effect of implementing hs-cTn testing on the diagnosis and outcomes of patients with kidney impairment is uncertain.

## Methods

High-Sensitivity Troponin in the Evaluation of Patients With Acute Coronary Syndrome (High-STEACS) was a stepped-wedge, cluster-randomized clinical trial that evaluated the use of a hs-cTnI assay in consecutive patients with suspected acute coronary syndrome across 10 hospitals (NCT01852123) (Supplement 1; eAppendix in Supplement 2).^3^ The trial was conducted in accordance with the Declaration of Helsinki and with the approval of the Scotland Research Ethics Committee, the Public Benefit and Privacy Panel for Health and Social Care, and each National Health Service Health Board. As randomization was at the hospital level, individual patient consent was not sought. Throughout the trial, cTnI was measured using contemporary and high-sensitivity assays (ARCHITECT*_STAT_*; Abbott Laboratories). Before use, results from the hs-cTnI assay were suppressed, and the contemporary assay (single threshold based on the coefficient of variation) guided care. Sites were then randomly assigned to early or late use of hs-cTnI testing, for which results from the contemporary assay were suppressed and care was guided by the hs-cTnI assay with sex-specific 99th percentile diagnostic thresholds.

Estimated glomerular filtration rate (eGFR) was calculated using the Chronic Kidney Disease Epidemiology Collaboration equation. Kidney impairment was defined as an eGFR of less than 60 mL/min/1.73 m^2^. In this prespecified secondary analysis, the primary outcome of subsequent type 1 or 4b myocardial infarction following the index presentation or cardiovascular death within 1 year was compared before and after implementation of the hs-cTnI assay in all patients with elevated hs-cTnI concentrations and in the subgroup of patients reclassified by hs-cTnI testing with normal contemporary troponin concentrations, as stratified by eGFR (<60/≥60 mL/min/1.73 m^2^) using Cox models that were adjusted for age, sex, phase, hospital site (random effect), seasonality, presentation date, diabetes, ischemic heart disease or cerebrovascular disease, hs-cTnI concentrations, and deprivation status. Statistical analysis was performed in R, version 3.6.1 (R Foundation). A 2-sided *P* value of less than .05 was considered to indicate statistical significance.

## Results

Across both phases, hs-cTnI concentrations were elevated in 10 111 of 46 927 patients (22%; mean [SD] age, 71 [15] years; 4853 women [48%]), of whom 4220 (42%) had kidney impairment. The proportion of patients with elevated hs-cTnI concentrations increased as kidney function declined, from 10% (1911 of 19 763) at an eGFR of 90 or greater to 66% (1171 of 1766) at an eGFR of less than 30 mL/min/1.73 m^2^ (*P* < .001) (Figure, A). In contrast, the proportion of patients with type 1 myocardial infarction decreased from 74% (1261 of 1709) to 35% (328 of 934) (*P* < .001) (Figure, B).

**Figure.**
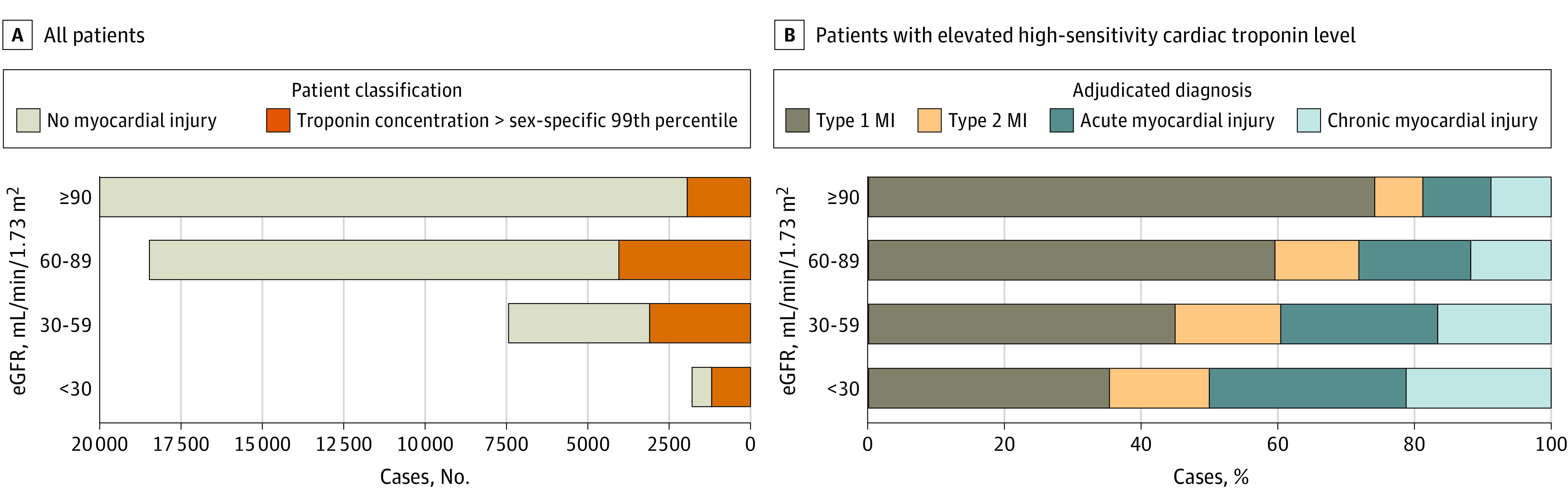
Myocardial Injury and Myocardial Infarction (MI) in Patients With Kidney Impairment A, Number of patients with high-sensitivity cardiac troponin concentrations above and below the sex-specific 99th percentile across the entire study population (n = 46 927) according to estimated glomerular filtration rate (eGFR). B, Frequency of adjudicated diagnoses in patients with high-sensitivity cardiac troponin concentrations above the sex-specific 99th percentile (n = 10 111), according to eGFR.

Following the use of hs-cTnI testing, the proportion of patients with an elevated troponin increased from 37% (1386 of 3721) to 47% (2503 of 5359) and from 13% (1918 of 14 686) to 16% (3610 of 23 161) in those with and without kidney impairment, respectively (*P* < .001 for both). Despite identifying more patients at risk, the rate of subsequent type 1 or 4b myocardial infarction or cardiovascular death at 1 year in all patients with an elevated hs-cTnI concentration was similar before and after use in those with (25% vs 24%; adjusted hazard ratio [aHR], 1.00; 95% CI, 0.85-1.18) and without kidney impairment (13% vs 11%; aHR, 0.89; 95% CI, 0.73-1.08) (Table). Similarly, the primary outcome was unchanged in the subgroup of reclassified patients in those with (18% vs 15%; aHR, 1.04; 95% CI, 0.62-1.74) and without kidney impairment (12% vs 11%; aHR, 1.17; 95% CI, 0.69-1.96).

**Table.** Outcomes of Patients With hs-cTnI Concentrations Above the Sex-Specific 99th Percentile, Grouped by Study Phase and eGFR

Characteristic	No. (%)						
	eGFR, mL/min/1.73 m^2^						Overall
	<60			≥60			
	Overall	Before	Use	Overall	After	Use	
No. of participants	4220	1717	2503	5891	2281	3610	10 111
Primary outcome							
MI or cardiovascular death^a^	1016 (24)	426 (25)	590 (24)	686 (12)	293 (13)	393 (11)	1702 (17)
Secondary outcomes							
MI^a^	313 (7)	129 (8)	184 (7)	357 (6)	168 (7)	189 (5)	670 (7)
Unplanned revascularization^b^	116 (3)	44 (3)	72 (3)	283 (5)	114 (5)	169 (5)	399 (4)
All-cause death	1500 (36)	662 (39)	838 (34)	808 (14)	353 (16)	455 (13)	2308 (23)
Death of cardiovascular causes	785 (19)	330 (19)	455 (18)	367 (6)	143 (6)	224 (6)	1152 (11)
Death of cardiac causes	630 (15)	261 (15)	369 (15)	294 (5)	113 (5)	181 (5)	924 (9)
Hospital admission for heart failure	601 (14)	250 (15)	351 (14)	396 (7)	195 (9)	201 (6)	997 (10)
Ischemic stroke	95 (2)	47 (3)	48 (2)	100 (2)	50 (2)	50 (1)	195 (2)
Safety end points							
Major hemorrhage^c^	43 (1)	21 (1)	22 (1)	57 (1)	22 (1)	35 (1)	100 (1)
Unplanned hospital admission at 30 d^d^	1158 (27)	537 (31)	621 (25)	1805 (31)	820 (36)	985 (27)	2963 (29)
Noncardiovascular death	715 (17)	332 (19)	383 (15)	440 (8)	210 (9)	230 (6)	1155 (11)

Abbreviations: eGFR, estimated glomerular filtration rate; hs-cTnI, high-sensitivity cardiac troponin; MI, myocardial infarction.

^a^ Subsequent type 1 or type 4b MI.

^b^ Defined as urgent or emergency percutaneous coronary intervention or coronary artery bypass grafting from discharge to 1 year later.

^c^ Bleeding Academic Research Consortium type 3 or type 5.

^d^ Excludes type 1 or type 4b MI.

## Discussion

While the frequency of elevated hs-cTnI concentrations increased 6-fold as kidney function declined from an eGFR of 90 or greater to less than 30 mL/min/1.73 m^2^, the proportion attributable to type 1 myocardial infarction halved. Although hs-cTnI is effective at enabling the early rule out of myocardial infarction in patients with kidney impairment,^4,5^ use did not improve outcomes in patients with elevated levels whether they had kidney impairment or not. The reasons for this are complex. Two-thirds of patients with kidney impairment and elevated hs-cTnI concentrations had a diagnosis other than type 1 myocardial infarction. In the absence of evidence from randomized trials, there is little guidance to inform clinical decisions for this heterogeneous group. Moreover, for those with kidney impairment and type 1 myocardial infarction, the available evidence is largely extrapolated from clinical trials in patients with broadly normal kidney function.^6^ Further research is therefore needed to convince clinicians of the safety and efficacy of these treatments in patients with kidney impairment. A limitation of this study is that it was not possible to discriminate between acute and chronic kidney injury. While both are associated with cardiovascular risk, these conditions are distinct. Following the use of hs-cTnI testing in clinical practice, 1 in 2 patients with kidney impairment had an elevated troponin concentration, but these were less likely due to myocardial infarction, and outcomes did not improve.

## Notes

Supplement 1. Trial protocol

Supplement 2. eAppendix. Implementation of high-sensitivity cardiac troponin in patients with renal impairment

Supplement 3. Nonauthor collaborators. The High-STEACS Investigators

Supplement 4. Data sharing statement
