# Supplementary material for: Use of High-Sensitivity Cardiac Troponin in Patients With Kidney Impairment: A Randomized Clinical Trial
Source: JAMA Intern Med. 2021 Jun 7;181(9):1237–9. doi: 10.1001/jamainternmed.2021.1184 (PMC8185626; doi:10.1001/jamainternmed.2021.1184)

Statistical Analysis Plan

High-STEACS

High-Sensitivity Troponin in the Evaluation of patients with Acute Coronary Syndrome (High-STEACS): a randomised controlled trial

**Version:**	1.0
**Authors:**	Catriona Keerie, Christopher Weir
**Date:**	16th May 2018
**Chief Investigator:**	Professor Nicholas L Mills

Document Version History

Version Number	Reason for Update	Updated By:	Date
draft 1.0	Creation of new SAP	Catriona Keerie	08 Nov 2016
1.0_22Nov2016	Revision of SAP	Nicholas Mills	22 Nov 2016
1.0_24Nov2016	Amendments following project team review	Catriona Keerie	24 Nov 2016
1.0_08Dec2016	Amendments following project team review	Catriona Keerie	08 Dec 2016
1.0_01Mar2017	Amendments following project team review and alignments with HiSTORIC SAP	Catriona Keerie	01 Mar 2017
1.0_04Apr2017	Modification of analysis population definitions to match available data	Christopher Weir	06 Apr 2017
1.0_21Apr2017	Addition of GRACE score and season definitions	Christopher Weir	21 Apr 2017
1.0 05May2017	Chief Investigator review	Christopher Weir	05 May 2017
1.0 12Oct2017	Final Chief Investigator and TSC review	Christopher Weir	12 Oct 2017
1.0 19Nov2017	Edits following final review and removal of Sinj analysis population	Christopher Weir	19 Nov 2017
1.0 29Nov2017	Further draft for TSC review	Christopher Weir	29 Nov 2017
1.0 20Mar2018	Update following TSC review	Christopher Weir	20 Mar 2018
1.0 26Apr2018	Final redrafting following TSC feedback	Christopher Weir	26 Apr 2018
1.0 16May2018	Clinical team input and protocol v7.0 changes	Christopher Weir	16 May 2018
1.0	Version for signature	Christopher Weir	16 May 2018

Signatures

**Chief Investigator**<br>**Prof Nicholas L Mills**<br>BHF Centre for Cardiovascular Sciences<br>University of Edinburgh	Signature	16 May 2018<br>Date
**Trial Statistician**<br>**Prof Christopher Weir**<br>Edinburgh Clinical Trials Unit<br>Usher Institute of Population Health Sciences & Informatics<br>University of Edinburgh	Signature	16 May 2018<br>Date

1 Table of Contents

1 Table of Contents 3

2 List of Abbreviations 4

3 Introduction 5

3.1 Study Design 5
3.2 Study Objectives 6
3.3 Original Hypothesis 6

4 Statistical Methods Section from the Protocol 6

5 Overall statistical principles 6

5.1 SAP Objectives 6
5.2 General Statistical Principles 7
5.3 Analysis Populations 7

6 List of analyses 8

6.1 Recruitment 8
6.2 Baseline Characteristics 9
6.3 Management and discharge 10
6.4 Primary Outcome 11
6.4.1 Event summaries 11
6.4.2 Primary analysis: logistic regression mixed modelling with covariate adjustment 11
6.4.3 Sensitivity analysis: logistic regression mixed model with interaction term 12
6.4.4 Secondary analysis: Unadjusted logistic regression mixed model 12
6.4.5 Secondary analysis: Time to event summaries 12
6.4.6 Secondary analysis: Cox proportional hazards modelling 13
6.5 Secondary outcomes 13
6.6 Safety analyses 13
6.7 Long term follow-up 14

7 Derived variables 14

7.1 Timed endpoints 14
7.2 GRACE score 14
7.3 Troponin sampling 14
7.4 Primary endpoint 14

8 Missing data 14

9 Validation 15

10 Data sharing 15

11 References 15

Appendix 1 16

Appendix 2 18

2 List of Abbreviations

A&E	Accident and emergency
ACE	Angiotensin converting enzyme
AIC	Akaike's Information Criterion
ARB	Angiotensin receptor blocker
BARC	Bleeding Academic Research Consortium
CABG	Coronary artery bypass graft
CI	Confidence interval
CONSORT	CONsolidated Standards of Reporting Trials
DAPT	Dual anti-platelet therapy
ECTU	Edinburgh Clinical Trials Unit
eGFR	estimated Glomerular Filtration Rate
FA	Full analysis
GRACE	Global Registry of Acute Coronary Events
Inj	Myocardial injury
IHD	Ischaemic heart disease
LBBB	Left bundle branch block
MI	Myocardial infarction
NInj	No myocardial injury
PCI	Percutaneous coronary intervention
PPI	Proton pump inhibitor
Q1	Lower quartile
Q3	Upper quartile
RInj	Reclassification with myocardial injury
RMI	Reclassification with myocardial infarction
SAP	Statistical analysis plan
SD	Standard deviation
SIMD	Scottish Index of Multiple Deprivation
SOP	Standard operating procedure
URL	Upper reference limits

3 Introduction

3.1 Study Design

In this prospective stepped wedge cluster randomised controlled multi-centre trial, there will be three 6-month study phases: *validation*, *randomisation*, and *implementation*. The ARCHITECT *STAT* high-sensitivity troponin I assay will be run in parallel with the existing contemporary sensitive troponin I assay (standard care) throughout the trial. During the *validation* phase, all sites will report results from the contemporary assay and the diagnostic threshold for myocardial infarction will remain unchanged. This phase will provide baseline information on patients with suspected acute coronary syndrome for each site. In the *randomisation* phase, participating centres will be randomised 1:1 to introduce the high-sensitivity assay or continue with the contemporary assay and existing diagnostic thresholds (standard care). Centres will be matched by number of admissions and type of health care provider (secondary and tertiary care). Finally, in the *implementation* phase, all centres will implement the high-sensitivity assay for at least 6 months (see diagram below).

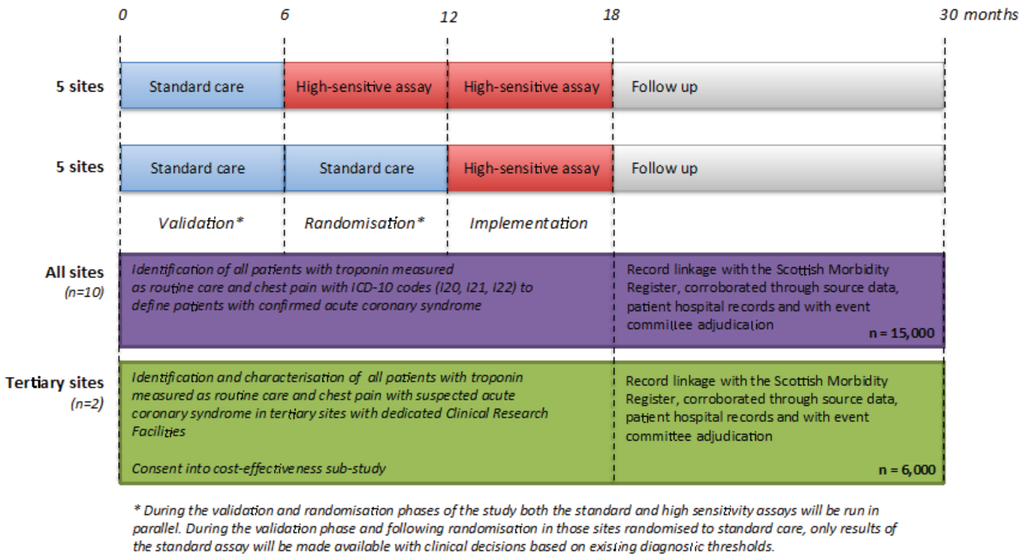

The use of a cluster-randomised design permits the inclusion of all patients with suspected acute coronary syndrome, prevents selection bias and limits the potential for confusion amongst clinicians who would otherwise be required to simultaneously assess patients using different diagnostic criteria for acute myocardial infarction. It is anticipated that approximately 23,800 patients will be included in total across the three phases of the trial.

This document details the criteria to be used for the definition of the analysis populations and the statistical methodology for analysis for the High-STEACS trial. It has been compiled according to the Edinburgh Clinical Trials Unit (ECTU) standard operating procedure (SOP) "ECTU_SOP_ST_04 Statistical Analysis Plans" and has been written based on information contained in the study protocol version 7.0.

3.2 Study Objectives

To determine whether use of a high-sensitivity cardiac troponin I assay to identify the 99th percentile upper reference limit as the diagnostic threshold for myocardial infarction is appropriate in patients with suspected acute coronary syndrome.

3.3 Original Hypothesis

Implementation of the ARCHITECT *STAT* high-sensitive troponin I assay will reduce recurrent myocardial infarction and cardiovascular death at one year in patients with suspected acute coronary syndrome.

4 Statistical Methods Section from the Protocol

This section repeats the statistical analysis section from the protocol to allow any necessary changes from the planned methods to be clearly presented.

The analysis will take account of the cluster-randomised nature of the stepped wedge design. The primary end-point of 12-month event-free survival (reinfarction and cardiovascular death) will be compared before and after implementation of the high-sensitivity assay using a logistic regression generalized linear mixed model. Primary endpoint survival times will also be summarised descriptively using Kaplan-Meier survival curves and analysed formally using a Cox proportional hazards model incorporating a site level random effect and fixed effects for assay and covariate effects. The proportional hazards assumption will be verified by plotting log-cumulative hazard versus log-time for the high sensitivity and standard care troponin assay groups. Model validity will be further explored using plots of Cox-Snell and Martingale residuals.

Secondary efficacy endpoint (i), duration of stay, will be analysed using Kaplan-Meier survival curves and a Cox proportional hazards model as above, with death in hospital being treated as a censoring event. Secondary efficacy endpoints (ii)-(vii) and safety outcomes will be analysed using the same methods as used for the primary efficacy endpoint.

5 Overall statistical principles

5.1 SAP Objectives

The objective of this SAP is to describe the statistical analyses contributing to the final report and primary publication(s) of the High-STEACS study. This SAP does not address the analyses required for the proposed sub-studies and exploratory analyses.

The planned analyses will be performed in a staged manner, according to the availability of the relevant study data. In particular, analyses of the adjudicated MI and RMI populations (defined

in Section 5.3) and the adjudicated diagnosis (Section 6.2) will be deferred and will not be reported at the same time as the other analyses. Further phasing of the reporting may also be implemented according to the subsets of the analyses listed in this SAP which would be required for scheduled conference presentations and peer reviewed journal publications.

5.2 General Statistical Principles

Hypothesis tests will be performed at the two-sided 5% significance level. Categorical variables will be summarised with the number and percentage of subjects falling in each category. Continuous variables will be summarised using the mean, median, standard deviation (SD), lower quartile (Q1), upper quartile (Q3), minimum and maximum values. P-values will be stated to two significant figures after the decimal point, with exceptionally small p-values being reported as "<0.0001".

In all time to event analyses, observations will be censored at the earlier of study end date or, if recorded, the date of migration out of Scotland. Event times will be measured from the arrival date and time of the index presentation.

5.3 Analysis Populations

Eligible sites are secondary and tertiary hospitals in Scotland which use the ARCHITECT platform. The number of subjects who were screened (patients in whom cardiac troponin was requested) and included will be reported. All patients whom the treating physician requested a cardiac troponin measurement for suspected acute coronary syndrome who have valid paired cardiac troponin I measurements on both the contemporary and high-sensitivity assay during the index presentation will be included in the study. If data are missing on whether suspected acute coronary syndrome was the reason for the cardiac troponin measurement request, the patient will be included in the study. Readmissions during follow-up are not be eligible for entry to the study as a new participant. Patients who are not resident in Scotland will be excluded as follow-up via routinely collected sources will not capture the primary outcome for these individuals. Reasons for non-inclusion will be summarised, as will the number of participants completing the study. All included patients with evidence of myocardial necrosis (high-sensitivity cardiac troponin concentration >99th centile using sex-specific upper reference limits [URL] on presentation or subsequent testing) will be identified and two investigators will independently review all clinical information, including non-invasive and invasive investigations from presentation to 30 days. Patients will be classified as having type 1, type 2, type 4a, type 4b and type 5 myocardial infarction, or having myocardial injury according to the universal definition of myocardial infarction. Type 1 myocardial infarction will be defined as myocardial necrosis in the context of an isolated presentation with suspected acute coronary syndrome with chest pain or evidence of myocardial ischemia on the electrocardiogram. Patients with symptoms or signs of myocardial ischemia due to increased oxygen demand or decreased supply (e.g. tachyarrhythmia, hypotension or anaemia) and myocardial necrosis will be classified as type 2 myocardial infarction. Peri-procedural myocardial infarction type 4a will be defined in patients with a rise of 20% in cardiac troponin concentration if values are elevated and are stable or falling prior to angioplasty; type 4b will be defined in patients with stent thrombosis documented at coronary angiography or at autopsy; type 5 will be defined in patients following cardiac surgery when cardiac troponin concentrations are >10 × 99th percentile during the first 48 hours from a normal baseline. Myocardial injury will be defined as evidence of myocardial necrosis in the absence of any clinical features of myocardial ischaemia. Any discrepancies will

be resolved by the adjudication of a third independent reviewer. The adjudication committee will be blinded to study phase.

Details regarding the interpretation of troponin results and the classification of patients based on these values are provided in Appendix 1.

Full analysis (FA) population
Patients who have paired cardiac valid troponin I measurements on both the contemporary and high-sensitivity assay within 24 hours of the index presentation arrival date/time.

Myocardial injury (Inj) and myocardial infarction (MI) populations
Patients with myocardial injury (Inj) are defined as those with elevated cardiac troponin I concentrations identified by the contemporary assay (≥50 ng/L in NHS Lothian; 0.04 ng/mL in NHS Greater Glasgow & Clyde). Within this population, patients with myocardial infarction (MI) will consist of those with an adjudicated diagnosis of type 1 or type 4b myocardial infarction.

Reclassification with myocardial injury (RInj) and myocardial infarction (RMI) populations
Patients reclassified with myocardial injury (RInj) are defined as those with elevated cardiac troponin I concentrations identified using the high-sensitivity assay (>99th percentile sex-specific upper reference limit [URL], 34 ng/L in men and 16 ng/L in women) in whom concentrations were below the diagnostic threshold on the contemporary assay. Within this population, patients reclassified with myocardial infarction (RMI) will consist of those with an adjudicated diagnosis of type 1 or type 4b myocardial infarction.

No myocardial injury (NInj) population
Patients with cardiac troponin I concentrations ≤99th percentile sex-specific URL using the high-sensitivity assay.

All analyses and data manipulations will be carried out using SAS [1].

6 List of analyses

6.1 Recruitment

CONSORT type [2] flowchart, adapted for stepped wedge design, including details of:

- Number of sites
- Number of sites dropping out (if any)
- Issues with intervention delivery or timing within the sites (if any).
- Number of participants recruited overall and within each site
- Number of participants recruited within each intervention/control condition, within each site
- Number of participants providing complete follow-up data within each intervention/control condition, within each site
- Number of participants included in the primary analysis within each intervention/control condition, within each site

A schematic representation of the actual study design will be reported with hospital sites, randomised groups, and intervention start points.

Sites were paired based on expected number of presentations and one site randomised to early implementation and the other to late implementation. For pragmatic reasons (shared lab

facilities), the Vale of Leven and Royal Alexandra Hospital, Paisley were grouped and randomised together. This enabled implementation of the high-sensitivity assay to occur on the same date at both sites and allowed the same lab processes to be followed at both sites.

6.2 Baseline Characteristics

Baseline participant and site characteristics will be summarised before and after implementation of the ARCHITECT *STAT* high-sensitivity troponin I assay for each study site and for the FA. Baseline characteristics will also be summarised for the Inj and RInj populations, MI and RMI populations, and the NInj populations. No formal hypothesis testing of the baseline characteristics will be performed.

The site characteristics considered will include:

- Hospital size
- Location (Glasgow & Clyde, Lothian)

The participant characteristics considered will include:

- Age (years)
- Sex (male/female)
- Cardiovascular risk factors
    - Smoking status (current/ex/never)
    - Hyperlipidaemia (yes/no)
    - Diabetes mellitus (yes/no)
    - History of hospitalisation with heart failure
- Past medical history
    - Ischaemic heart disease (IHD)
    - Myocardial infarction (MI)
    - Cerebrovascular disease
    - Previous percutaneous coronary intervention (PCI)
    - Previous coronary artery bypass graft (CABG)
- Scottish Index of Multiple Deprivation (SIMD) (quintile)

- Medical therapy on presentation (yes/no)
    - Aspirin
    - Clopidogrel
    - Prasugrel
    - Ticagrelor
    - Dual antiplatelet therapy (DAPT)[1]
    - Angiotensin converting enzyme (ACE) inhibitor / angiotensin receptor blocker (ARB)
    - Beta blocker
    - Statin
    - Oral anticoagulant
    - Calcium channel blockers
    - Nicorandil

[1] As indicated by aspirin and any other of three anti-platelet medications (clopidogrel, prasugrel or ticagrelor)

- Ivabradine
- Spironolactone
- Loop diuretic
- Proton pump inhibitor (PPI)

Clinical presentation (Index admission)

- Time interval, symptom onset to troponin sampling (hours)
- Time interval, presentation to troponin sampling (minutes)
- Time interval, troponin sampling at presentation to first repeat sample (minutes)
- Primary symptom (chest pain, dyspnoea, syncope, palpitation, other)
- GRACE score [3]
- Measurements at initial assessment:
    - Heart rate (bpm)
    - Systolic / diastolic blood pressure (mmHg)
    - 12-lead electrocardiogram
        - Rhythm (SR, AF/Flutter, SVT, VT/VF, other)
        - Ischaemic changes (yes/no)
        - ST elevation (yes/no)
        - ST depression (yes/no)
        - T wave inversion (yes/no)
        - LBBB new (yes/no)
        - LBBB old (yes/no)

Haematology and clinical chemistry

- Haemoglobin
- Creatinine
- eGFR

Troponin concentrations

- Presentation high-sensitivity cardiac troponin concentration (ng/L)
- Maximum high-sensitivity cardiac troponin concentration (ng/L)
- Proportion of patients with serial measurements (2 or more) during index presentation (%)

Adjudicated diagnosis

- Type 1 myocardial infarction (STEMI, NSTEMI)
- Type 2 myocardial infarction
- Type 4a myocardial infarction
- Type 4b myocardial infarction
- Type 5 myocardial infarction
- Myocardial injury
- Other clinical diagnosis

6.3 Management and discharge

Management during the index hospitalisation and therapies at discharge will be summarised before and after implementation of the ARCHITECT *STAT* high-sensitivity troponin I assay for each study site and overall for the FA population. These characteristics will also be summarised

for the Inj and RInj populations, MI and RMI populations, and the NInj populations. The following variables will be reported:

- Duration of stay, hours
- Coronary angiography (yes/no)
- Percutaneous coronary revascularisation (yes/no)
- Surgical coronary revascularisation (yes/no)
- Medical therapy from electronic discharge summary
    - Aspirin
    - Clopidogrel
    - Prasugrel
    - Ticagrelor
    - Dual antiplatelet therapy (DAPT)
    - Angiotensin converting enzyme (ACE) inhibitor / angiotensin receptor blocker (ARB)
    - Beta blocker
    - Statin
    - Oral anticoagulant
    - Calcium channel blockers
    - Nicorandil
    - Ivabradine
    - Spironolactone
    - Loop diuretic
    - Proton pump inhibitor (PPI)

6.4 Primary Outcome

6.4.1 Event summaries

FA, Inj, RInj, MI, RMI, NInj populations
Myocardial infarction (type 1 or type 4b) following index presentation or cardiovascular death at 1 year will be summarised (n, %) by study site and overall before and after implementation of the ARCHITECT *STAT* high-sensitivity troponin I assay.

6.4.2 Primary analysis: logistic regression mixed modelling with covariate adjustment

RInj population
The primary outcome will be compared before and after implementation of the high-sensitivity assay using a logistic regression generalised linear mixed model following the model structure described in Hussey and Hughes (2007) [4] (see Appendix 2). The effect of implementation of the high-sensitivity assay will be presented as an odds ratio and its 95% confidence interval (CI).

The event rate will be modelled using a logistic mixed-effects regression model, adjusting for hospital site, season (Spring, Summer, Autumn, relative to Winter[2] as the reference category), time of patient presentation since start of study (days), and an indicator variable for whether the high-sensitivity assay has been implemented or not. Hospital site will be fitted as a random

[2] Spring (March, April, May) Summer (June, July, August) Autumn (September, October, November) Winter (December, January, February) as per the UK Met Office definitions http://www.metoffice.gov.uk/learning/learn-about-the-weather/how-weather-works/when-does-spring-start

effect and age, sex and SIMD 2016 quintile will be included as fixed patient-level covariates in the model. The age variable will be centred on the mean age in the RInj population.

Time of patient presentation since start of study (the date on which the validation phase started in the first study site) will be initially modelled as a linear term; alternatives will be considered if the data do not support the linearity assumption. Time since start of study will be centred on the mid-point of the study recruitment period and scaled to a range similar to that of the age covariate in order to minimise model convergence issues. Should the primary analysis modelling fail to converge, one covariate at a time will be omitted from the model until convergence is achieved. Covariates will be considered for omission in the order sex, age, SIMD 2016.

In order to accommodate the closure of the Western Infirmary Glasgow (WIG) and Victoria Infirmary Glasgow (VIG) during the study, and the subsequent redirection of patients in their catchment areas to the new Queen Elizabeth University Hospital (QEUH) on the site of the Southern General Hospital (SGH), patients will be analysed according to the site at which they were treated. QEUH will be considered a continuation of the Southern General Hospital site. The assumptions in this approach will be investigated in a sensitivity analysis in which SGH and QEUH patients from the WIG or VIG catchment areas are excluded from the analysis.

The above modelling will be repeated for the FA, Inj, MI, RMI, NInj analysis populations to provide supporting information. For each population the odds ratio and 95% CI will be displayed graphically by site using a forest plot (where feasible, based on the number of participants and outcome events in each site for a given analysis population).

6.4.3 Sensitivity analysis: logistic regression mixed model with interaction term

RInj population

A sensitivity analysis will be performed in which additional terms will be included in the primary analysis model fitted in section 6.4.2, according to the extension outlined in Hemming *et al* [5], to fit a site by intervention interaction random effect. This is intended to aid interpretation of the primary analysis results, with regard to intervention-effect heterogeneity across sites. This will be repeated for the FA, Inj, MI, RMI, NInj analysis populations as supporting information. Prospective simulation studies suggest that model fitting with this additional interaction term have a 3% to 21% risk of non-convergence of the model, depending on the scenario simulated. The covariate-omission strategy described in section 6.4.2 will be used to maximise the chances of convergence.

6.4.4 Secondary analysis: Unadjusted logistic regression mixed model

RInj population

A secondary analysis not adjusting for patient-level covariates will also be performed. This will be repeated for the FA, Inj, MI, RMI, NInj analysis populations as supporting information.

6.4.5 Secondary analysis: Time to event summaries

FA, Inj, RInj, MI, RMI, NInj populations

Primary outcome survival times will be summarised descriptively before and after implementation of the high-sensitivity assay using Kaplan-Meier survival curves (with

accompanying 95% confidence intervals). Differences before and after implementation will be tested using a log-rank test stratified by site.

6.4.6 Secondary analysis: Cox proportional hazards modelling

FA, Inj, RInj, MI, RMI, NInj populations

Survival times will be analysed formally using a Cox proportional hazards model following a similar form to the logistic regression model of Section 6.4.2, fitting hospital site as a random effect and age (centred on the analysis population mean), sex and SIMD quintile as patient-level fixed effects. The effect of implementing the high-sensitivity assay will be estimated by the hazard ratio and its 95% confidence interval. The proportional hazards assumption will be verified by plotting log-cumulative hazard versus log-time before and after implementation of the high-sensitivity assay. Model validity will be further explored using plots of Cox-Snell and Martingale residuals. Given the difficultly in assessing hazard proportionality in the presence of a hospital site random effect, the above checks will be performed for each hospital site separately. Should the proportional hazards assumption be invalid, survival analysis will be restricted to the descriptive Kaplan-Meier curves and log-rank tests of section 6.4.5 only.

6.5 Secondary outcomes

The following secondary outcomes will be analysed using the methods outlined in sections 6.4.1, 6.4.2, 6.4.3 and 6.4.4:

- Type 1 or type 4b myocardial infarction following the index presentation
- Unplanned coronary revascularisation after discharge
- Cardiovascular death
    - Any cardiovascular death
    - Cardiovascular death – Cardiac subset
    - Cardiovascular death – Non-cardiac subset
- All-cause death
- Hospitalisation for heart failure
- Ischaemic stroke

6.6 Safety analyses

Safety outcomes will be summarised before and after implementation of the ARCHITECT STAT high-sensitive troponin I assay for the FA population. Safety characteristics will also be summarised for the Inj, RInj, MI, RMI, NInj populations. The following safety outcomes will be reported:

- Major haemorrhage[3]
- Unplanned hospitalization within 30 days excluding acute coronary syndrome
- Non-cardiovascular death

[3] BARC Type 3 and BARC Type 5

6.7 Long term follow-up

Long term follow up at 3 and 5 years will capture relevant efficacy and safety outcomes. Analysis and reporting of long term follow-up data will be presented separately to the planned analyses documented here.

7 Derived variables

7.1 Timed endpoints

- Time from symptom onset to troponin sampling (minutes)
- Time from symptom onset to time of presentation (minutes)
- Time from presentation to troponin sampling (minutes)
- Time from troponin sampling at presentation to first repeat sample (minutes)

7.2 GRACE score

- The GRACE score and calculation of the predicted probability of in-hospital death [6] will be determined for all patients with elevated high-sensitivity cardiac troponin I concentrations above the sex-specific 99th centile upper reference limit.

7.3 Troponin sampling

- Presentation high-sensitivity cardiac troponin concentration (ng/L)
- Maximum high-sensitivity cardiac troponin concentration (ng/L)
- Proportion of patients with serial measurements (2 or more) during index presentation (%)

7.4 Primary endpoint

Myocardial infarction (type 1 or type 4b) following index presentation or cardiovascular death at 1 year will be captured as 2 separate variables from the adjudication database and a Yes/No flag will be created to summarise the primary endpoint.

8 Missing data

It is anticipated that SIMD quintile data will be missing in a small proportion of patients, for example due to the creation of additional postal code areas for new housing developments. As SIMD quintile is a key covariate for adjustment in the primary analysis, any missing SIMD quintile will be substituted, using single imputation, with the modal value observed on the other patients treated at the same study site.

No other imputation of missing data will be performed, apart from the handling of high sensitivity and contemporary assay troponin values and their measurement times as described in Appendix 1.

9 Validation

The following will be done by a second statistician:

1. Separate programming and checking of primary outcome results and conclusions.

2. The statistical report will be read and sense-checked.

10 Data sharing

A file, or set of files, containing the final data will be prepared, along with a data dictionary. These will be made available to the Chief Investigator within the Safe Haven secure area at the end of the analysis phase.

11 References

1. SAS® Institute Inc. SAS for Windows. SAS Institute Inc.: Cary, NC, U.S.A

2. Campbell MK, Piaggio G, Elbourne DR, Altman DG, for the CONSORT Group. Consort 2010 statement: extension to cluster randomised trials. BMJ 2012; 345:e5661.

3. Granger CB, Goldberg RJ, Dabbous O, Pieper KS, Eagle KA, Cannon CP et al. Predictors of hospital mortality in the Global Registry of Acute Coronary Events. Arch Intern Med 2003; 163:2345-2353.

4. Hussey MA, Hughes JP. Design and analysis of stepped wedge cluster randomized trials. Contemporary Clinical Trials 2007; 28:182-191.

5. Hemming K, Taljaard M, Forbes A. Analysis of cluster randomised stepped wedge trials with repeated cross-sectional samples. Trials 2017;18:101.

6. Anderson F, FitzGerald G. Methods and formulas used to calculate the GRACE Risk Scores for patients presenting to hospital with an acute coronary syndrome. Copyright 1998-2014, Center for Outcomes Research, University of Massachusetts Medical School.

Appendix 1

1) Units
Contemporary troponin results: ng/mL (both Lothian and Glasgow)
High-sensitivity troponin results: ng/L (Lothian) or pg/mL (Glasgow) which are equivalent.

2) Thresholds:
Contemporary troponin assay
Glasgow: <0.04 ng/mL equivalent to 40 ng/L
Lothian: <0.05 ng/mL equivalent to 50 ng/L

High-sensitivity troponin assay for both Lothian and Glasgow
Women: 16 ng/L
Men: 34 ng/L

NOTE: for data analysis
** high-sensitivity troponin results are reported as integers to clinicians. Therefore, any value ≥16.5 ng/L for women and ≥34.5 ng/L for men will be "positive" for myocardial injury.

3) Presentation high-sensitivity troponin result
-where the initial high-sensitivity troponin is non-numeric (ie. IS= insufficient sample or TC= test cancelled), take next result as "presentation" result

4) Duplicate troponin results with same date/time
If there are duplicate troponin results with same date and time:
-take first result
-If non-numeric (ie. IS= insufficient sample or TC= test cancelled, then take next result)
-this does not apply to troponin results with time 00:00 (see below)

5) Sample time with 00:00 erroneously
- These results might have been inadvertently excluded because time 00:00 is before the presentation time
-Solution: For these patients, use result date/time from variable RESULTDATE.

6) Defining Reclassification subgroups
The following rule will be applied in a hierarchical fashion:

	C	HS
Inj	+	
RInj	-	+
Ninj	-	-

7) With regard to missing paired troponins
some patients with unpaired troponin may still be included
For example:

	C	HS
cannot classify [exclude]*	missing	+ (< 100 ng/L)
Inj	missing	+ (≥ 100 ng/L)
NInj	missing	-
Inj	+	missing
cannot classify [exclude]*	-	missing
cannot classify [exclude]*	missing	missing

*these troponin values will be excluded but patient will remain with other paired results.

8) Definition of presentation sample
First valid troponin result (contemporary or high-sensitivity).

9) Definition of serial sampling
Serial samples will be defined as two or more valid and clinically reported troponin results within 24 hours. Therefore, in the Validation phase this will be defined as two or more valid contemporary troponin result within 24 hours and in the Implementation phase, two valid high-sensitivity troponin result within 24 hours. Proportion of patients with serial sampling will be calculated for all patients and in the subset of patients who presented within 6 hours of chest pain onset.

Appendix 2

Statistical model for the analysis of the primary outcome

The statistical model for the analysis of the primary outcome (Section 6.4.2) is formally defined as follows:

The event rate μ_{ijk} in site *i*, season *j,* time of presentation since start of study T_k for patient k (i=1,…,10; j=1,2,3,4; k = 1, …) will be modelled in the linear component of the logistic regression as:

$$\mu + \alpha_i + \beta_j + \gamma T_k + \theta X_{ik}$$

where μ is the overall mean event rate on the logit scale;
α_i is a random effect for site *i*, $\alpha_i \sim N(0,\tau^2)$;
β_j is the fixed effect log-odds ratio for season *j*;
γ is the linear fixed effect for time of presentation since start of study;
θ is the fixed effect log-odds ratio for the high-sensitivity assay treatment;
X_{ik} is a (0/1) variable to indicate whether the high-sensitivity assay is implemented in site *i* for patient *k*.

Key patient-level covariates of age, sex and SIMD will also be included as fixed effects.

***High-sensitivity T*roponin in the *E*valuation of patients with *A*cute *C*oronary *S*yndrome (*HighSTEACS*): A randomised controlled trial**

Chief Investigator: Prof Nicholas L Mills

On behalf of the High-STEACS Trial Investigators

Funders: British Heart Foundation
Abbott Diagnostics

Sponsor: University of Edinburgh
NHS Lothian Health Board

ClinicalTrials.gov Identifier: NCT01852123

REC Number: 12/SS/0115

Trial Start Date: 10 June 2013
Trial Finish Date: 02 March 2018
Trial Report Date: 02 September 2018

Correspondence Address and Main Centre:

Professor Nicholas L Mills
Room SU225, Chancellor's Building, University of Edinburgh, Royal Infirmary, Little France, Edinburgh, EH16 4SB, UK.
Tel: +44 (0) 131 242 6517
Fax: +44 (0) 131 242 6379 Email: nick.mills@ed.ac.uk

COORDINATING CENTRE

**Chief Investigator**	**Co-Sponsor Representative**
Prof Nicholas L Mills<br>Room SU225, Chancellor's Building, University of Edinburgh, Royal Infirmary, Little France, Edinburgh, EH16 4SB, UK.<br>Tel: +44 (0) 131 242 6517<br>Fax: +44 (0) 131 242 6379<br>Email: nick.mills@ed.ac.uk	Marise Bucukoglu<br>The Queen's Medical Research Institute<br>47 Little France Crescent<br>Edinburgh<br>EH16 4TJ<br>Tel: 0131 242 9262<br>Fax: 0131 242 9447<br>Email: marise.bucukoglu@ed.ac.uk
**Trial Manager**	**Trial Statistician**
Chris Tuck<br>Edinburgh Clinical Trials Unit, University of Edinburgh<br>Level 2, Nine Edinburgh Bioquarter, Edinburgh, EH16 4UX<br>Tel: +44 (0) 131 651 9907<br>Email: chris.tuck@ed.ac.uk	Prof Christopher Weir<br>Edinburgh Clinical Trials Unit, University of Edinburgh<br>Level 2, Nine Edinburgh Bioquarter, Edinburgh, EH16 4UX<br>Tel: +44 (0) 131 651 9957<br>Email: christopher.weir@ed.ac.uk

PARTICIPATING SITES

**NHS Lothian**<br>**(Royal Infirmary of Edinburgh, Western General Hospital, St John's Hospital)**<br>**Principal Investigator**<br>Prof Nicholas L Mills<br>Room SU225, Chancellor's Building, University of Edinburgh, Royal Infirmary, Little France, Edinburgh, EH16 4SB, UK.<br>Tel: +44 (0) 131 242 6517<br>Fax: +44 (0) 131 242 6379<br>Email: nick.mills@ed.ac.uk	**NHS Greater Glasgow and Clyde**<br>**(Glasgow Royal Infirmary, Western Infirmary and Gartnavel General Hospital, Southern General Hospital, Victoria Infirmary, Paisley Alexandra Hospital, Inverclyde Royal Hospital, Vale of Leven General Hospital)**<br>**Principal Investigator**<br>Professor Colin Berry<br>Department of Cardiology, Western Infirmary, G11 6NT<br>Tel: +44 (0) 141 211 2000<br>Email: colin.berry@glasgow.ac.uk
Sub study 1 RIE & WGH<br>Prof Nicholas L Mills<br>Room SU225, Chancellor's Building, University of Edinburgh, Royal Infirmary, Little France, Edinburgh, EH16 4SB, UK.<br>Tel: +44 (0) 131 242 6517<br>Fax: +44 (0) 131 242 6379<br>Email: nick.mills@ed.ac.uk	Sub study 1 RAH<br>Dr Iain Findlay<br>Department of Cardiology, Royal Alexandra Hospital, PA<br>Tel:<br>Email: iain.findlay@ggc.scot.nhs.uk
Sub study 2 & 3 RIE<br>Prof Nicholas L Mills<br>Room SU225, Chancellor's Building, University of Edinburgh, Royal Infirmary, Little France, Edinburgh, EH16 4SB, UK.<br>Tel: +44 (0) 131 242 6517<br>Fax: +44 (0) 131 242 6379<br>Email: nick.mills@ed.ac.uk	Sub study 1 GRI<br>Dr Donogh Maguire<br>Emergency Department, Glasgow Royal Infirmary, 84 Castle Street, Glasgow, G4 0ET<br>Tel: 0141 211 4000<br>Email: donogh.maguire@ggc.scot.nhs.uk

Contents:

Section		Pages
1.	Protocol Approval	5
2.	Investigator Statement	6
3.	Summary	7
4.	Background	8
5.	Study Objectives	14
6.	Trial Design	15
7.	Data Analysis and Statistical Considerations	21
8.	Expected Results	27
9.	Safety Reporting	28
10.	Study Monitoring	29
11.	Ethical and Regulatory Issues	30
12.	References	31
13.	Appendices	33

1 Protocol Approval

***High-sensitivity T*roponin in the *E*valuation of patients with *A*cute *C*oronary *S*yndrome (*HighSTEACS):* A randomised controlled trial**

Signatures

Prof Nicholas Mills<br>Chief Investigator	______________________<br>Signature	____________<br>Date
Prof Christopher Weir<br>Trial Statistician	______________________<br>Signature	____________<br>Date

2 Investigator Statement

***High-sensitivity T*roponin in the *E*valuation of patients with *A*cute *C*oronary *S*yndrome (*HighSTEACS):* A randomised controlled trial**

I agree to conduct the study according to this protocol, the principles of International Conference on Harmonisation Tripartite Guidelines for Good Clinical Practice (ICH GCP) and the applicable regulatory requirements. Any changes in procedure will only be made if necessary to protect the safety, rights or welfare of the patients.

I agree to take responsibility for the conduct of the study and ensure that all other staff involved are adequately informed about the protocol and amendments and their study related duties and functions.

Signatures

______________________________ _______________
Signature of Investigator Date

____
Name of Investigator (please print)

3 Summary

We have recently demonstrated that lowering the diagnostic threshold for troponin is associated with a halving in the rate of recurrent myocardial infarction and death in patients redefined with myocardial infarction. We here propose to determine whether further lowering the diagnostic threshold for myocardial infarction using a novel high-sensitivity cardiac troponin assay will continue to improve outcomes in patients with suspected acute coronary syndromes. If increased sensitivity does not impinge on the specificity of the diagnosis of myocardial infarction, then these assays will improve patient outcome through better targeting of therapies for coronary heart disease. However, if increased sensitivity leads to poor specificity, then patients may be misdiagnosed and given inappropriate cardiac medications with potentially detrimental outcomes. In 10 secondary and tertiary care hospitals across Scotland, we will undertake a cluster randomised controlled trial of the implementation of a novel high-sensitivity troponin assay in approximately 15,000 patients presenting with suspected acute coronary syndromes. The primary end-point will be the one-year rate of death or recurrent myocardial infarction. This will establish whether the introduction of high-sensitivity assays into routine clinical practice is detrimental or beneficial to patient management and outcome; a fundamental and critical assessment for the modern definition of acute myocardial infarction.

4 Background

Coronary heart disease is the commonest cause of death in the United Kingdom. There are 124,000 patients admitted with acute myocardial infarction *per annum* in the UK, and despite significant improvements in the management of coronary disease over the last 20 years, the 30-day mortality remains unacceptably high.

The risks associated with myocardial infarction lead to large numbers of patients self-presenting or being referred by primary care to the Emergency Department with undifferentiated chest pain of suspected cardiac origin. These patients are responsible for 5 per cent of all presentations to the Emergency Department and 40 per cent of unplanned hospital admissions [Goodacre et al., 2005]. This therefore represents a major burden on scarce health care resource. Approaches to improve the accurate identification of patients with acute myocardial infarction would therefore be welcome and potentially of major benefit to patients and the NHS.

Definition of myocardial infarction

Myocardial infarction has, for many years, been defined by the clinical history, electrocardiogram and a rise in serum creatine kinase as evidence of myocardial necrosis. Recent improvements in the sensitivity of assays to detect myocardial necrosis required a more precise definition. The Global Task Force for the Universal Definition of Myocardial Infarction and the National Academy of Clinical Biochemistry now recommend that any detectable increase in cardiac troponin above the 99th percentile of a healthy reference population should be used to diagnose myocardial infarction [Thygesen et al., 2007; Morrow et al., 2007]. This statement was based on expert consensus rather than evidence from randomised controlled trials and the guidelines have not been universally adopted. The diagnostic threshold recommended by the Global Task Force has been implemented in fewer than 1 in 3 hospitals in Europe [Collinson et al., 2011a] and none in North America where the Food and Drug Administration have yet to approve the use of high-sensitivity troponin assays.

The use of out dated diagnostic thresholds for troponin continues to be widespread and lowering this threshold remains a highly contentious issue in clinical practice [Mills et al., 2011c; Bauer et al., 2009]. Progressively lowering the threshold of plasma troponin concentration in order to define increasing numbers of patients with myocardial infarction may not be appropriate because of the potential to reduce specificity and increase false positive diagnoses of myocardial infarction, with raised troponin levels occurring in a range of common presentations including septicaemia, pulmonary embolism, and cardiac and renal failure [McFalls et al., 2011]. This could lead to an increase in unnecessary hospital admissions, misplaced investigations and treatment, and may be detrimental to patient care.

The Global Task Force addressed the potential for more sensitive troponin assays to reduce clinical specificity by proposing a classification system for myocardial infarction based on the aetiology of myocardial necrosis [Thygesen et al., 2007]. Type I myocardial infarction is defined as a spontaneous myocardial infarction with ischemia due to a primary coronary event such as plaque erosion and/or rupture, fissuring, or dissection. Type II myocardial infarction is defined as myocardial ischemia secondary to either increased oxygen demand or decreased supply (e.g. coronary artery spasm, coronary embolism, anaemia, arrhythmia, hypertension or hypotension).

Differentiating between primary and secondary causes of myocardial injury is not straightforward and therefore this classification system has not been adopted in clinical practice.

Implementation of a contemporary sensitive troponin assay in NHS Lothian
Following improvements in assay performance, we replaced our conventional troponin assay with a contemporary sensitive troponin assay. The validation and subsequent implementation of the ARCHITECT *STAT* troponin I assay provided us with an opportunity to assess the impact of a more sensitive assay on the management and clinical outcome of patients with suspected acute coronary syndrome [Mills et al., 2011a].

Sensitive troponin assays improve clinical outcomes
In our cohort of 2,092 patients, lowering the diagnostic threshold from 200 to 50 ng/L with the ARCHITECT *STAT* troponin I assay increased the incidence of myocardial infarction by 29%. During the validation phase of this study only values at or above the diagnostic threshold of 200 ng/L from the previous generation of assay were reported to clinicians. Perhaps surprisingly patients with troponin concentrations greater than 200 ng/L had better clinical outcomes than those with small undisclosed increases in plasma troponin concentrations of 50-200 ng/L. These latter patients were 2-3 times more likely to have an adverse outcome in comparison to those with more marked elevation in troponin.

Implementation of the sensitive assay and lowering the diagnostic threshold to 50 ng/L was associated with a more than halving of death and recurrent myocardial infarction in patients who would otherwise not have been classified as having sustained an acute myocardial infarction. In this group of patients improvements in clinical outcome were associated with more referrals to a cardiologist (74% versus 44%), and greater use of dual anti-platelet therapy (58% *versus* 27%) and coronary angiography (46% *versus* 20%)($P<0.001$ for all). To our knowledge, this is the first time that the introduction of a new assay into clinical practice has changed management and reduced cardiovascular mortality. Whilst important, these observations were derived from a single tertiary centre using a troponin assay that is not able to achieve the necessary precision to use the 99th percentile of a normal reference population as the diagnostic threshold.

Lowering the threshold for diagnosis of myocardial infarction
The Global Task Force for the Universal Definition of Myocardial Infarction and the National Academy of Clinical Biochemistry recommends the 99th percentile as the diagnostic threshold where assay precision can be demonstrated with a coefficient of variation (CV) of ≤10% at this concentration. Whilst the ARCHITECT *STAT* troponin I assay was clearly superior to our previous conventional troponin assay it was unable to meet these precision criteria and therefore we selected a diagnostic threshold above the 99th percentile where the CV is ≤10% in order to minimise the risk of false positive results and making an inappropriate diagnosis of myocardial infarction.

In exploratory analysis from the same cohort, we assessed the relationship between troponin concentration, assay precision and clinical outcome [Mills et al., 2011b; under revision]. We demonstrate that patients with troponin concentrations above the 99th percentile (12 ng/L according to manufacturer), but below our revised diagnostic threshold (50 ng/L), were more likely to be dead or readmitted with recurrent myocardial infarction compared to those with troponin concentrations in the normal range of <12 ng/L (13% versus 3%, adjusted OR 2.3, 95% CI 1.3-4.0, P=0.004; Figure 1). Although patients with troponin concentration 12 to 49 ng/L were older and more

likely to have established ischaemic heart disease or cardiovascular risk factors than those patients with a troponin concentration in the normal reference range, plasma troponin concentration remained an independent predictor of clinical outcome even after adjustment for these clinical characteristics and despite reduced assay precision below the diagnostic threshold (CV up to 28.9%). Our findings are consistent with recent studies of patients with acute coronary syndrome participating in randomized clinical trials where troponin concentrations above the 99th percentile measured from stored sample were associated with poorer outcomes [Eggers et al., 2009; Lindahl et al., 2010]. Importantly, our study population comprised consecutive "real-world" patients admitted with suspected cardiac chest pain rather than a homogeneous group of patients with a clinical diagnosis of acute coronary syndrome on optimal therapy.

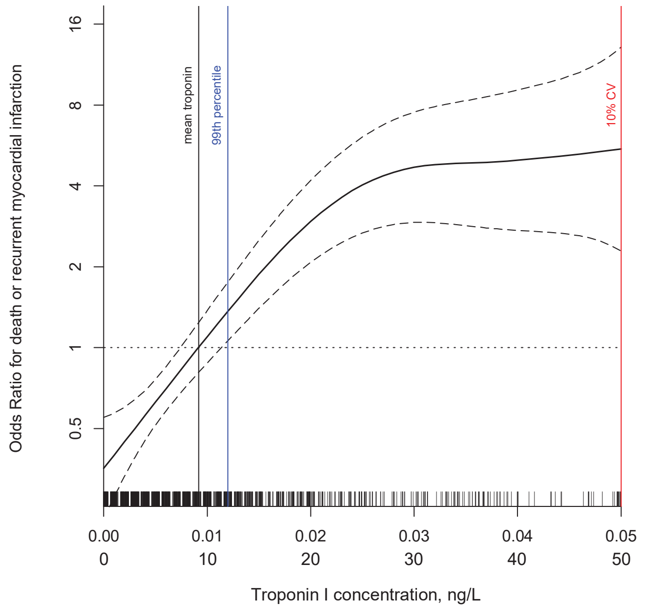

Figure 1**: **Association between cardiac troponin concentration and odds of death or recurrent myocardial infarction relative to odds at the mean troponin (9 ng/L, black line) among patients with troponin concentrations below the diagnostic threshold of 50 ng/L (red line). The 99th percentile for a healthy reference population is 12 ng/L (blue line). Estimates were obtained from a generalized additive model using a cubic smoothing spline (df=3, P=0.005 for non-linearity). A rug plot illustrates the density of the data for given value of troponin.

Lowering the diagnostic threshold to the 99th percentile will identify more patients at risk of recurrent events, but would potentially increase the number of patients diagnosed with myocardial infarction by a further 46% from 752 to 1,104 of 2,092 patients admitted with suspected acute coronary syndrome. This will have major implications for health care resources, public health targets, government statistics, and on the employment prospects and insurance policies of our patients. Will lowering the threshold further and reclassification of patients with suspected acute coronary syndrome as myocardial infarction alter clinical outcome?

In our original study patients admitted during the validation phase of our contemporary sensitive assay with an undisclosed troponin concentration of 50 to 200 ng/L had a high event rate of 39% at one year [Mills et al., 2011a]. In comparison patients with undisclosed troponin concentrations 12 to 50 ng/L are at lower risk with a one-year event rate of 13%. Therefore there may be less to gain from the reclassification and treatment of these patients for myocardial infarction. Additional discussion on this point is included in the attached manuscript under revision. The need for prospective randomised controlled trials to determine whether the use of high-sensitivity troponin assays with greater analytical precision at the 99th percentile will translate into improvements in clinical outcomes was highlighted by authorities in the field including the Editor of *Heart,* Dr Adam Timmis, during the open peer review of this manuscript.

Additional studies have further shown the benefits of serial changes in high sensitive troponin and early diagnosis of myocardial infarction [Keller et al 2011c] providing further evidence of the role of troponin in accurate and rapid assessment of patients with acute coronary syndrome and possibly facilitating rapid discharge.

Impact of sensitive troponin assays on clinical specificity

Many clinicians are concerned that the use of progressively lower diagnostic thresholds will result in patients being misdiagnosed with myocardial infarction and that small troponin elevations will also be caused by other pathologies. This may lead to inappropriate hospital admission, misdiagnosis, initiation of potentially detrimental therapies and poorer clinical outcomes for patients.

In a further analysis of patients admitted during the validation and implementation of our contemporary sensitive troponin I assay we identified consecutive patients in whom peak troponin concentrations were between 50 to 200 ng/L *irrespective of presenting symptom* (manuscript in preparation). Two independent cardiologists reviewed all patient records and classified patients according to the universal definition of myocardial infarction as type 1 (primary myocardial ischemia) or type 2 (secondary myocardial ischemia). Disagreements were resolved following in-depth case note and source file review until a consensus was achieved. We identified 922 patients with peak cardiac troponin concentrations between 50 to 200 ng/L during the 6-month validation (n=525; reported as troponin 'negative') and implementation phases (n=397; reported as troponin 'positive'). These patients were classified as having type 1 myocardial infarction in 28% (n=275) and type 2 myocardial infarction in 72% (n=666). So for every patient with a spontaneous type 1 myocardial infarction identified using a sensitive troponin assay we identified nearly three patients with secondary myocardial ischemia and a clear alternative clinical diagnosis.

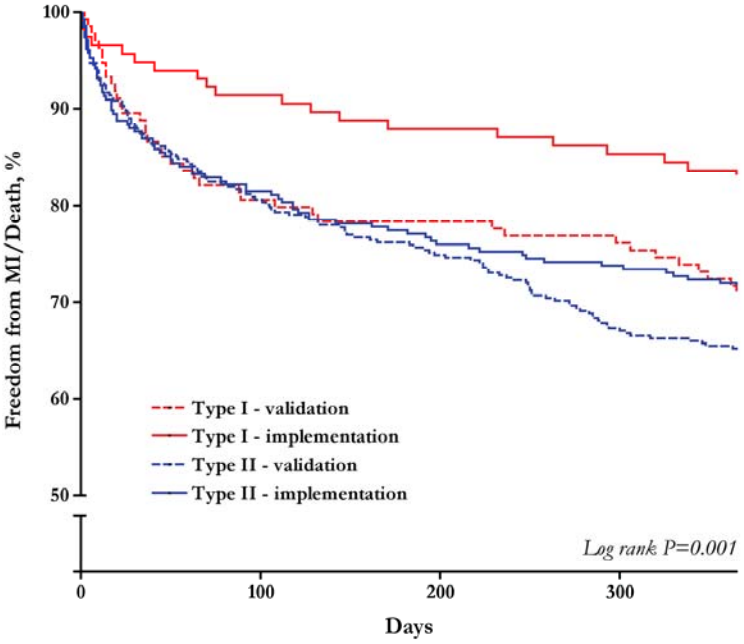

Figure 2. Survival free from death or recurrent myocardial infarction in patients with type 1 and type 2 myocardial infarction. Patients with undisclosed increases in cardiac troponin with type 1 or type 2 myocardial infarction had similar clinical outcomes during the validation period. Following implementation of the sensitive assay death and recurrent myocardial infarction was reduced in patients with type 1 myocardial infarction (31% vs 17%, P=0.009), but did not change in patients with type 2 myocardial infarction (36% vs 29%, P=0.09).

Interestingly although patients with type 2 myocardial infarction had worse clinical outcomes compared to patients with type 1 myocardial infarction (Figure 2), this was primarily driven by death (28%) rather than recurrent myocardial infarction (6%). Following implementation of the sensitive assay, referrals to cardiology increased in patients with type 2 myocardial infarction (19% to 34%, P<0.001) as did requests for

cardiac echocardiography (12% to 23%, P<0.001) and the use of coronary angiography (1% to 6%, P<0.001). Despite more extensive investigation in patients with type 2 myocardial infarction, clinical outcomes (recurrent myocardial infarction and all cause mortality) were similar whether they were managed as troponin 'negative' during the validation phase or troponin 'positive' following implementation. Reduced clinical specificity for primary myocardial infarction will be an even greater challenge with the next generation of high-sensitivity troponin assays and even lower diagnostic thresholds. As such, prospective studies with a formal assessment of cost-effectiveness are essential.

Conventional, contemporary sensitive, high-sensitivity and ultra-sensitive cardiac troponin assays

The use of ***conventional*** troponin assays lead to patients being classified as troponin 'negative' or 'positive' with clear therapeutic options, but assay imprecision required higher diagnostic thresholds with an increased risk of clinically important false negative results. The current generation of ***contemporary sensitive*** troponin assays improve the early diagnosis of myocardial infarction [Reichlin et al., 2009; Keller et al., 2009] and clinical outcome in patients with suspected acute coronary syndrome [Mills et al., 2011b]. Indeed in a recent randomised controlled trial of point-of-care testing in the emergency room (RATPAC trial) troponin measured on admission was as effective as a multiple biomarker strategy or serial measurements to rule out acute myocardial infarction [Collinson et al., 2011b].

At present few contemporary sensitive assays are able to achieve the recommended precision (co-efficient of variation <10%) to use the 99th percentile of a normal reference population as the diagnostic threshold in clinical practice. There is only one commercially available ***high-sensitivity*** assay troponin assay (high-sensitivity troponin T assay; Roche Diagnostics, Basel, Switzerland) that has been approved for clinical use in Europe. This assay has greater analytical sensitivity than contemporary sensitive assays and is able to detect cardiac troponin at lower concentrations such that troponin is measureable in about 50% of apparently healthy persons [Keller et al., 2011]. However, the high-sensitivity troponin T assay appears to have similar sensitivity and specificity for the diagnosis of acute myocardial infarction compared to current contemporary sensitive troponin I assays [Reichlin et al., 2009]. The latest development in assay technology involves magnetic nanoparticle-based immunoassays coupled with single-molecule counting and permits cardiac troponin to be accurately quantified in >98% of healthy persons at concentrations <1 ng/L [Sabatine et al., 2009]. These ***ultra sensitive*** assays are an interesting research tool, but the platform does not permit the rapid throughput necessary to make clinical decisions in the emergency department.

ARCHITECT STAT high-sensitive troponin I assay

The prototype ARCHITECT *STAT* high-sensitive troponin I assay (Abbott Diagnostics) is a two-step assay that offers increased precision for measuring very low plasma troponin concentrations and can quantify troponin concentrations in 98% of healthy persons (personal communication, Dr Fred Apple) with a limit of detection of 1 ng/L and 10% co-efficient of variation <5.5 ng/L [Matias et al., 2010]. Using this assay the mean±standard deviation concentration of a healthy reference population is 1.6±3.1 ng/L with the 99th percentile of 16 ng/L. Whilst this prototype assay is likely to be marketed as a 'high-sensitivity' assay it is able to detect troponin across the complete reference range of a healthy population with sensitivity comparable to the leading ultra-

sensitive research assay [Sabatine et al., 2009]. This assay therefore has the sensitivity and precision to report absolute measurements of troponin and a normal reference range, rather than an arbitrary threshold based on limited assay performance. Critically it can be delivered within 60 minutes of sample collection using the existing ARCHITECT platform, which is already used by more than fifty NHS hospitals across the United Kingdom and in all of the participating centres in the proposed trial.

High-sensitivity cardiac troponin and the early rule out of myocardial infarction

Until recently NICE guidelines have recommended measuring cardiac troponin on presentation and 10-12 hours after the onset of symptoms to coincide with the peak in circulating troponin concentration (Skinner et al., 2010). This allows the assessment of infarct size, and will minimise the risk of missing a small myocardial infarct. However, it requires the majority of patients to be admitted to hospital for serial testing placing pressure on already overcrowded emergency departments and medical admissions units.

Recently, NICE reviewed the available evidence comparing high-sensitivity cardiac troponin assays with standard troponin assays for the early rule out of myocardial infarction [NICE Diagnostic Guidance D15 available at https://www.nice.org.uk/guidance/dg15]. They conclude that the use of high-sensitivity troponin on presentation and at 3 hours was both clinically effective and a cost-effective strategy compared to admission of patients for standard troponin testing at 10-12 hours, and recommended the use of high-sensitivity cardiac troponin assays in the National Health Service.

A number of areas of uncertainty were identified. First, whilst most studies have shown that high-sensitivity cardiac troponin measured at 3 hours has good negative predictive value for myocardial infarction (Keller et al., 2011, Cullen et al., 2013, Reichlin et al., 2012), these studies were based on retrospective analysis of small cohorts. Second, many studies selected low-risk patients and therefore the findings may not be generalizable across all patients presenting with suspected acute coronary syndrome (Cullen et al., 2013, Collinson et al., 2012a). Third, ***no study used the high-sensitivity assay as the reference standard***, and therefore the adjudication of the diagnosis of myocardial infarction was based on peak troponin testing using a standard assay. This will overestimate both the sensitivity and negative predictive value of the high-sensitivity assay measured at 3 hours (Shah et al., 2013). Finally, few studies have not been large enough to address important subgroups of patients, such as those who present early and within 3 hours of the onset of their symptoms.

In view of these uncertainties, NICE diagnostic guidance recommended careful audit of patient outcomes following implementation of high-sensitivity assays and early rule out pathways.

5 Study Objectives

In both secondary and tertiary care hospitals across Scotland, we will undertake a stepped wedge cluster randomised controlled trial of the implementation of the ARCHITECT STAT high-sensitive troponin I assay. The main objective of this study is to determine whether identifying the 99th percentile of a normal reference population as the diagnostic threshold for myocardial infarction is appropriate in patients with suspected acute coronary syndrome. There is the potential for both major patient benefit as well as harm in this approach. If the increased sensitivity does not impinge on the specificity of the diagnosis of myocardial infarction, then the next generation of high-sensitivity assays will confer large healthcare benefits and improve patient outcome through better targeting of therapies for coronary heart disease. However, if the increased sensitivity leads to poor specificity, then patients will be misdiagnosed with myocardial infarction, inappropriately given cardiac medications and potentially have adverse and detrimental outcomes. This may also lead to mismanagement of the patient due to a lack of recognition and treatment of the true underlying cause of their symptoms.

In addition, there will be an exploratory objective to look at efficacy and safety of the implementation of the high-sensitive troponin I assay stratified by age, sex, diagnostic threshold (sex-specific, uniform), diagnostic classification (type 1-5 myocardial infarction, myocardial injury), and renal function. We will also examine the association between patient characteristics, clinical diagnoses and all primary and secondary outcomes in the study population using a variety of statistical (including Bayesian) and machine learning approaches.

5.1 Original Hypothesis

Implementation of the ARCHITECT STAT high-sensitive troponin I assay will reduce myocardial infarction or death at one year in patients with suspected acute coronary syndrome.

6 Trial Design

Design: Prospective stepped wedge cluster randomised controlled trial

There will be three equal 6-month study phases in the stepped wedge design: validation, randomisation, and implementation (see section 13). The ARCHITECT STAT high-sensitive troponin I assay will be run in parallel with the existing contemporary sensitive troponin I assay (standard care) throughout the study. During the validation phase, the diagnostic threshold for myocardial infarction will remain unchanged. This phase will provide baseline information on patients with suspected acute coronary syndrome for each site. In the randomisation phase, participating centres will be randomised 1:1 to introduce the high-sensitivity assay or continue with the existing diagnostic thresholds (standard care). Centres will be matched by number of admissions and type of health care provider (secondary and tertiary care). Thereafter all centres will implement the high-sensitivity assay for at least 6 months.

The stepped wedge design allows each participating centre to act as its own control and permits adjustment for seasonal differences in the incidence of acute myocardial infarction and temporal changes in clinical outcome due to advances in clinical practice. It is neither practical nor advantageous to randomise individual patients in this trial. The use of a cluster-randomised design permits the inclusion of *all patients* with suspected acute coronary syndrome, prevents selection bias and limits the potential for confusion amongst clinicians who would otherwise be required to simultaneously assess patients using different diagnostic criteria for acute myocardial infarction. We will also assess the appropriateness of sex-specific thresholds using the high-sensitivity assay prior to the start of the study.

Following a recommendation from the trial steering committee the 6-month implementation phase will be extended for those sites randomised to early implementation until all sites have completed the study protocol. This will provide additional information on the impact of seasonality on the primary end-point.

Setting: Multi-centre trial in 10 acute secondary and tertiary hospitals throughout Scotland (see Appendix).

Primary end-point: Event-free survival (myocardial infarction or cardiovascular death) at one year

Secondary efficacy end-points:
i) Duration of stay
ii) Myocardial infarction
iii) Unplanned coronary revascularisation
iv) Cardiovascular death (any; cardiac; non-cardiac)
v) All-cause death
vi) Hospitalization for heart failure
vii) Ischaemic stroke

Secondary safety end-points:
i) Major and minor haemorrhage
ii) Recurrent hospitalization excluding acute coronary syndrome
iii) Cardiovascular death excluding acute coronary syndrome
iv) Non-cardiovascular death

An increase in the correct identification of patients with myocardial infarction is likely to lead to improved outcomes as we have demonstrated in our previous study [Mills *et al*, 2011a]. However, we will also need to monitor for an increase in false positive diagnoses of myocardial infarction. The two principal safety hazards of a misdiagnosis of myocardial infarction are likely to relate to excess bleeding events (especially during the index admission) and a failure to investigate and treat a potentially serious alternative diagnosis. These hazards will be captured by specifically looking for bleeding events, and recurrent hospitalizations or deaths due to conditions not attributable to index or recurrent myocardial infarctions. We plan to follow up the study population at 3 and 5 years to capture relevant efficacy and safety outcomes.

Troponin assays:
The ARCHITECT STAT high-sensitive troponin I assay will be compared with the previous generation ARCHITECT *STAT* assay that is currently used for routine clinical testing in each of the 10 participating centres. All participating centres currently report the lowest concentration where the assay coefficient of variation is ≤10% when assessed under local laboratory conditions in accordance with international guidelines [Thygesen et al, 2007].

ARCHITECT STAT troponin I assay (standard care)
Limit of detection 10 ng/L
10% co-efficient of variation 42 ng/L (Greater Glasgow) and 50 ng/L (Lothian)
Diagnostic threshold 42 ng/L (Greater Glasgow) and 50 ng/L (Lothian)

ARCHITECT STAT high-sensitive troponin I assay
Limit of detection 1 ng/L
10% co-efficient of variation <5 ng/L
Proposed diagnostic threshold 16 ng/L (99th percentile of healthy reference population)

During the ***validation period*** both assays will be run in parallel and all sites will continue to report cardiac troponin using the standard assay and existing diagnostic thresholds. This period will permit the ARCHITECT STAT high-sensitive troponin I assay to be evaluated on each ARCHITECT platform within all of the participating centres. Analytical precision will be determined using a series of quality control standards and pooled serum.

Prior to randomisation ***sensitivity and specificity*** for the diagnosis of myocardial infarction will be determined in the cohort of patients admitted to the Royal Infirmary Edinburgh and Western Infirmary Glasgow. Two independent cardiologists will review clinical data including troponin concentrations measured using the standard assay, whilst unaware of troponin concentrations from the high-sensitivity assay. Patients will be classified as acute myocardial infarction, unstable angina, cardiac chest pain due to non-coronary pathology (e.g. tachyarrhythmia), non-cardiac chest pain (clear alternative diagnosis), or symptoms of unknown origin. Disagreements will be resolved following in-depth case note and source file review and adjudicated by a third cardiologist. We will also explore whether high-sensitivity cardiac troponin is useful in the diagnosis or rule out of other conditions associated with myocardial injury (e.g. pulmonary embolism).

For the high-sensitivity assay we will systematically evaluate the impact of the following variables on sensitivity and specificity: i) a sex-specific normal reference range and diagnostic thresholds [Apple et al., 2010], ii) absolute change in troponin

concentration (admission to 6 or 12 hours), iii) relative change in troponin concentration (admission to 6 or 12 hours) [Reichlin T et al., 2011]. The normal reference range and diagnostic threshold for the trial will be agreed across all sites prior to randomisation and sensitivity analysis (i-iii) will be used to guide clinical advice issued during the introduction of the high-sensitivity assay at each participating centre.

Study population:
At each participating centre, we will identify *all patients* with suspected acute coronary syndrome who have cardiac troponin I measured as part of their routine clinical assessment. The Community Health Index (CHI) number, date and time of sample, presence or absence of chest pain, and absolute troponin concentration will be extracted from the ARCHITECT database in each participating centre. All patients with a history of suspected acute coronary syndrome as recorded by the requesting clinician who have valid paired cardiac troponin I measurements on both the contemporary and high-sensitivity assay during the index presentation will be allocated a unique study number and will comprise the study population. Patients who are not resident in Scotland will be excluded as follow-up via routinely collected sources will not capture the primary outcome for these individuals. Excess clinical samples that would otherwise be discarded (surplus) will be anonymously stored for future evaluation and audit of biomarkers. The results of these assays will not be used to alter clinical management. All samples will be anonymised and linked by a unique non identifiable ID.

One of the major strengths of this approach is that we will identify *all patients* with suspected acute coronary syndrome, rather than limiting our findings to a selected group who may not be representative of the broad range of patients presenting to acute NHS hospitals.

Data collection and record linkage:
CHI is a population register containing details of all Scottish residents registered with a General Practitioner and will be used to link all data sources. The ***Scottish Morbidity Record (SMR)*** will be used to identify the discharge diagnosis of all study participants including whether patients were classified as having unstable angina (ICD-10 codes I20) or myocardial infarction (I21 and I22). These data are routinely collected by the Information Services Division (ISD) of NHS National Services Scotland and are considered some of the best health service data in the world combining high quality, consistency, national coverage and the ability to link data to allow individual patient-based analysis and follow up. ISD Scotland data quality is assessed regularly with the accuracy and completeness of coding compared to source documents demonstrating that ICD-10 codes for coronary heart disease (I20-25) are 94.2% accurate and 99.2% complete.

As in our previous studies [Mills *et al,* 2011a; Mills *et al*, 2011b], additional clinical information will be obtained through the TrakCare software application (InterSystems Corporation, Cambridge, MA, USA); an ***Electronic Patient Record (EPR)*** system used in Lothian for 6 years and in all participating centres prior to the start of the trial. Programmers at NHS Lothian and Edinburgh University will develop a standardised *pro forma* for the assessment of patients with chest pain to replace the current generic admission form that will be implemented across all participating sites. This *pro forma* will include data fields that the admitting or discharging clinician will be required to complete, with the majority of data fields populated from drop-down menus. The following data will be captured: time of onset of symptoms, time of hospitalisation,

patient demographics (e.g. age and sex, cardiovascular risk factors, medical therapy on admission), GRACE score, heart rate, blood pressure, management in the Emergency Department, referral to cardiology, and discharge location. Any change to medical therapy will be extracted from the patients' standardised electronic discharge summary. 12-lead electrocardiograms will be obtained from a digital archive, the ***MUSE Cardiology Information System*** (GE Healthcare Clinical Systems Ltd., Herts, UK) in all participating centres. Reports from diagnostic coronary angiography, percutaneous and surgical coronary revascularisation will be extracted from the ***TOMCAT database*** (Cardiovascular Information Management System, Philips, Amsterdam, Netherlands) in the tertiary centres for NHS Greater Glasgow & Clyde (Golden Jubilee National Hospital, NHS Golden Jubilee National Hospital) and NHS Lothian (Royal Infirmary of Edinburgh). The use of a dedicated admission *pro forma* and identical platforms (ARCHITECT, TrakCare, MUSE, TOMCAT) for routine data collection in all participating centres will ensure a high degree of standardisation across sites and minimise missing data.

In the two largest tertiary centres in Scotland with excellent clinical research infrastructure (Royal Infirmary of Edinburgh and Western Infirmary of Glasgow) and the largest district general hospital (Royal Alexandra Hospital, Paisley), dedicated research nurses will ***validate routine data*** sources. Patients will be identified and located from the ARCHITECT platform on a daily basis. All patients with chest pain in whom troponin was measured on admission or 6-12 hours after symptom onset will be identified. Clinical characteristics, risk factors, investigations and management will be obtained from hospital records during the admission.

Sub-study 1: Clinical- and cost-effectiveness of high-sensitivity troponins

The costs of a high-sensitivity troponin assay are small and its introduction may reduce duration of stay, unnecessary diagnostic investigations, recurrent admissions, and deaths. In contrast, the more sensitive assay may reduce specificity and increase the use of unnecessary cardiac investigations and treatments. It is therefore essential that cost-effectiveness analysis will measure the incremental cost per quality adjusted life year (QALY) gained using the high-sensitivity assay, compared to the standard assay.

Patients admitted to the two largest tertiary centres in Scotland (Royal Infirmary of Edinburgh and Glasgow Royal Infirmary) and the largest district general hospital (Royal Alexandra Hospital, Paisley), will be asked to give consent for inclusion into a ***sub-study*** that will permit storage of plasma and serum and will require the completion of an EQ-5D health survey during the index admission and after 1 and 12 months of follow up.

In consented patients, excess serum (surplus) from routine clinical blood sampling on admission and 6 or 12 hours will be frozen and stored. Additional blood samples will be obtained between admission and 6 hours post presentation (maximum of 40 mL sample volume on no more than four blood draws). Plasma and serum will be prepared and stored for future analysis to quantify troponin, novel biomarkers of myocardial ischemia and for further biochemical profiling. A whole blood sample will be obtained for DNA extraction and genotyping. Strategies for the diagnosis and rule out of myocardial infarction will be explored in the sub-study and validated across the main study population where possible. To explore issues around the certainty of diagnosis, we will ask clinicians and patients to rate their perception of whether their chest pain is due to myocardial infarction before the results of troponin tests are available.

Patients can be co-recruited into other studies and included in our study population. Clinicians and other researchers will be informed of recruitment to the High-STEACS sub-study through the electronic patient record systems. This will allow researchers to take into consideration any additional burden placed on the patient through co-enrollment onto subsequent studies.

In situations where individuals have capacity to consent, but are unable to read the patient information sheet (e.g. visual impairment) or are unable to write, consent will be witnessed by an independent person and witness signature obtained to confirm that all information about the study was conveyed and that the patient gave their consent to take part.

Measurement of costs and outcomes:

Within trial economic analysis of resource costs and health outcomes will be conducted on an intention-to-treat basis. Efficacy and safety outcomes will be determined via record linkage with the Scottish Morbidity Register by the Information Services Division (ISD) Scotland. Fatal and non-fatal primary and secondary end-points will be verified through source data and patient hospital records.

Safety endpoints will be monitored throughout the main study and form part of routine reports. The Trial Management Team, the Trial Steering Committee (TSC) and the data monitoring committee (DMC) will oversee safety monitoring throughout the study.

Death from a cardiovascular cause will be defined as death from all cardiovascular causes and any death without another known cause [Wallentin et al, 2009]. Myocardial infarction will be defined according to international guidelines when there is evidence of myocardial necrosis in a clinical setting consistent with myocardial ischemia [Thygesen et al, 2007]. In brief, a diagnosis of myocardial infarction will be made where a rise and fall of cardiac troponin above the diagnostic threshold (standard care = 10% CV; intervention = 99th percentile of a reference population) is demonstrated in the presence of symptoms of myocardial ischemia, ECG changes consistent with myocardial ischemia, and/or evidence of new loss of viable myocardium from cardiac imaging. Major and minor haemorrhage will be defined from linked data using criteria based on the American College of Cardiology Foundation/American Heart Association Task Force on Clinical Standards [ACCF/AHA Task force, 2013].

In the sub-set of consented patients, we will determine one-year cumulative costs of in-patient investigation and management, and evaluate self-reported health at baseline and at 1 and 12 months of follow up using the EQ-5D health survey. Patients will complete the Seattle Angina Questionnaire at 1 and 12 months to capture patient reported outcome. The EQ-5D score, will be combined with survival times to enable estimation of QALYs for all patients up to one year following randomisation. Multiple imputation will be used to address missing values. Longer run modeling of incremental costs and health outcomes will estimate the distribution of costs and QALYs calculated over the expected patient lifetimes.

Sub-study 2: Patient reported outcomes

The high-sensitivity cardiac troponin assay may allow us to rule out myocardial infarction earlier and support the discharge of patients who have not had a myocardial infarction directly from the Emergency Department. In sub study 2 we will explore patient perceptions around discharge. Patients who have had myocardial infarction

ruled out as a possible diagnosis will be invited to participate in sub-study 2 (Appendix 3).

Consented patients will be visited in their own home (or a place of their choosing) approximately one week after discharge. In-depth interviews surrounding their experience of discharge from hospital following this episode of chest pain will be conducted and audiotaped. Follow up investigations ordered for these patients and their outcomes at 1 year will be recorded. This sub study will help guide the introduction of early discharge protocols into clinical practice by considering patient perceptions around discharge.

Sub-study 3: High-sensitivity cardiac troponin and the early rule out of myocardial infarction

In addition to the evaluation of the implementation of the high-sensitivity assay as part of the main study, we propose a sub-study to evaluate the efficacy and safety of a pathway to rule out myocardial infarction on presentation. During the final 6-months of the implementation phase of the main trial, sites will continue to follow the standard chest pain pathway with troponin testing at 12 hours using the high-sensitivity assay (standard care). Participating centres will then implement an early rule out pathway (intervention) based on National Institute of Clinical Excellence (NICE) [https://www.nice.org.uk/guidance/dg15] and European Society of Cardiology guidelines where troponin will be measured on presentation and myocardial infarction ruled out if troponin concentrations are <5 ng/L with further testing at 3 hours if indicated (***Appendix 4)***. Implementation will be stepped using a similar approach to the main trial over the next 6-month period. After this 6 month period we will continue to identify patients across all sites for a further 6 months.

7 Data Analysis and Statistical Considerations

The trial results will be reported in accordance with the CONSORT guidelines and, where possible, the clinical profile of non-recruited and ineligible patients will be recorded.

Statistical analysis:
The analysis will take account of the cluster-randomised nature of the stepped wedge design. The primary end-point of 12-month event-free survival (reinfarction and cardiovascular death) will be compared before and after implementation of the high-sensitivity assay using a logistic regression generalized linear mixed model. Primary endpoint survival times will also be summarised descriptively using Kaplan-Meier survival curves and analysed formally using a Cox proportional hazards model incorporating a site level random effect and fixed effects for assay and covariate effects. The proportional hazards assumption will be verified by plotting log-cumulative hazard versus log-time for the high sensitive and standard care troponin assay groups. Model validity will be further explored using plots of Cox-Snell and Martingale residuals.

Secondary efficacy endpoint (i), duration of stay, will be analysed using Kaplan-Meier survival curves and a Cox proportional hazards model as above, with death in hospital being treated as a censoring event. Secondary efficacy endpoints (ii)-(vii) and safety outcomes will be analysed using the same methods as used for the primary efficacy endpoint. The statistical methods will be documented in full in a detailed statistical analysis plan.Pre-specified exploratory subgroup analyses are planned to determine efficacy and safety stratified by age, sex, diagnostic threshold (sex-specific, uniform), diagnostic classification (type 1-5 myocardial infarction, myocardial injury), and renal function.

Exploratory data analyses will examine the association between patient characteristics, clinical diagnoses and all primary and secondary outcomes in the study population. Analyses will be conducted using a variety of statistical (including Bayesian) and machine learning approaches. In combination, these approaches will allow us to develop clinical decision support tools to aid in diagnosis, risk prediction and management of patients with suspected acute coronary syndrome.

Full methods for exploratory analyses will be documented in separate statistical analysis plans.

Power calculations:
Over a 12-month period (2008-2009), 2,092 patients were admitted at the Royal Infirmary of Edinburgh with chest pain and suspected acute coronary syndrome [Mills *et al*, 2011a; Mills *et al*, 2011b]. Of these, 1,383 patients had troponin concentrations below the current diagnostic threshold of 50 ng/L and 350 of these patients were found to have an undisclosed increase in plasma troponin within the range of 12-50 ng/L. At 12 months, 47/350 (13%) patients with an undisclosed troponin rise were dead or readmitted with myocardial infarction compared to 31/1,033 (3%) of those with normal troponin concentrations of <12 ng/L. Implementation of the previous generation of troponin assay reduced 12-month death and recurrent myocardial infarction from 39 to 21% (OR 0.42, 95%CI 0.24-0.84, P=0.013). We estimate that patients identified with

the high-sensitivity assay will derive similar benefit, but given the lower event rate in this group (13%), we have powered the prospective trial to detect a 3% absolute risk reduction.

In the proposed study, 10 sites will contribute patients during three 6-month phases: the validation phase (standard care), randomisation phase (standard care/intervention) and implementation phase (intervention). For each site we can calculate the difference in proportions, between standard care and intervention, of the primary outcome during the 12 months following hospital admission. This difference in proportions will be approximately normally distributed with a standard deviation that depends on the primary outcome event rate under standard care and the number of patients recruited by the site. ISD data for the financial year 2008-2009 were used to estimate site recruitment during each 6-month phase [Appendix 2]. Based on an average site recruitment of 525 patients with suspected acute coronary syndrome during each 6-month phase, a 2.1% absolute risk reduction would be detectable with 80% power, while the power would be over 95% for detection of a change in absolute risk of 3%.

Sub study 1: Clinical- and cost-effectiveness analysis

Statistical analysis:
Data from sub study 1 will allow evaluation of strategies for diagnosis of type 1 MI and for prediction of a major adverse cardiac event (MACE) at 30 days.

Type I MI diagnosis
12hr troponin will be used to define the gold-standard diagnosis of type I MI.
The sensitivity, specificity, and positive and negative predictive value of five strategies will be evaluated:

1. 3hr troponin >99th percentile
2. Admission troponin >99th percentile
3. 1 or 2
4. 1 or 2 plus a 20% increase (“delta”) from admission to 3hrs
5. 20% increase (“delta”) from admission to 3hrs

In addition, diagnostic performance of a range of admission and “delta” cut-points will be explored using ROC curve methodology.

Prediction of MACE at 30 days
The same strategies will be evaluated as for type I MI diagnosis.

Subgroup analysis
The performance of each diagnostic/prognostic strategy will also be described within the subgroups:

- Admission within 3hrs of onset (Yes/No)
- Age (over 65 years/not over 65 years)
- Pre-existing coronary disease (Yes/No)

Sample size:

Pilot data indicate that about 20% of the population under study will have a final diagnosis of type I MI based on the peak or 12 hr troponin concentration. Realistic

estimates for recruitment over a period of 12 months indicate that it will be possible to enrol up to 1500 participants in the sub-study. The table below indicates the precision with which the sensitivity and specificity could be estimated – as defined by 95% confidence interval width – for a range of overall sample sizes (1000-1500) and levels of sensitivity and specificity (95-99%).

Overall sample size	N with type I MI	Sensitivity	Sensitivity 95% CI width	N without type I MI	Specificity	Specificity 95% CI width
1000	200	95%	+/-3.0%	800	95%	+/-1.5%
1000	200	99%	+/-1.4%	800	99%	+/-0.7%
1500	300	95%	+/-2.5%	1200	95%	+/-1.2%
1500	300	99%	+/-1.1%	1200	99%	+/-0.6%

For the purposes of providing an overview, the confidence intervals considered use the normal approximation to the binomial distribution. In the actual study analysis, exact intervals will be used since the levels of sensitivity and specificity attained are expected to be close to 100% for some of the strategies being evaluated.
From analysis of pilot study data we will estimate the pairwise correlations among the troponin-based diagnostic strategies listed above. These will be used to inform further sample size calculations to determine the precision with which differences in sensitivity and/or specificity between a given diagnostic strategy and a reference strategy could be estimated in the sub-study.

Sub study 2: Patient reported outcomes

A mixed methods approach will be adopted for the analysis of data from sub study 2 (Appendix 3).

Sample size:
Eighty patients will be included in Sub study 2:

**10 female patients aged <u><</u>65 years**	**Patients selected from those discharged after the result of a troponin taken at 12 hours**
**10 female patients aged >65 years**	
**10 male patients aged <u><</u>65 years**	
**10 male patients aged >65 years**	
**10 female patients aged <u><</u>65 years**	**Patients selected from those discharged after the result of a troponin taken on presentation**
**10 female patients aged >65 years**	
**10 male patients aged <u><</u>65 years**	
**10 male patients aged >65 years**	

The numbers selected will help provide a range of views from the patient population, through this stratified sample. The object is to uncover the widest range of views held by the participants in the two patient groups. When no new meanings are emerging from the in-depth interviews, theoretical saturation will have been reached. It is anticipated that the proposed sample sizes will provide enough participants to capture all relevant themes. No formal power calculations are required for this qualitative approach. Qualitative research aims for depth rather than breadth. Based on previous qualitative studies the overall sample size will allow for exploration of similarities and differences within and between the patient groups.

Sub-study 3: High-sensitivity cardiac troponin and the early rule out of myocardial infarction

Statistical analysis
Primary efficacy end-point: Length of hospital stay, defined as the length of time from initial presentation to the Emergency Department until final discharge from hospital

Primary safety end-point: Type 1 or type 4b myocardial infarction or cardiac death after discharge and within 30 days of index presentation

Statistical analysis

The primary efficacy outcome will be analysed using a linear mixed-effects regression model, adjusting for hospital site, season, time of patient presentation since start of study (in days), and an indicator variable for whether the early rule out pathway has been actually implemented or not. Season will feature in the model as a four category factor variable, with three indicator dummy variables for Spring (March April May), Summer (June July August) and Autumn (Sept Oct Nov); relative to Winter (Dec Jan Feb), which is the reference category. Hospital site will be included as a random effect in the model, and the length of stay outcome will be log-transformed prior to analysis to reduce the influence of extreme outliers and ensure that the model assumptions are valid. Results will be expressed as geometric mean factors with two-sided 95% confidence intervals. It is possible that a very small proportion of patients with long hospital stays of over 3 months might not have been discharged by the time of the final analysis; in which case, these patients will be excluded from the primary efficacy analysis, but the number and percentage of these patients will be reported.

If the primary efficacy outcome analysis is statistically significant at the two-sided 5% level, then we will proceed to perform a non-inferiority analysis on the primary safety outcome. This will involve fitting a logistic mixed-effects regression model, adjusting for hospital site (as a random effect), season (defined as for the primary efficacy analysis), time of patient presentation since start of study (in days), and an indicator variable for whether the new intervention pathway has been implemented or not. A one-sided 95% confidence interval will be reported and a one-sided 5% significance threshold will be used. If the upper limit of the one-sided 95% confidence interval lies below the 0.5% non-inferiority margin; then non-inferiority of the intervention will be established. If non-inferiority is shown, then the primary safety outcome results will be assessed for superiority by interpreting the upper limit of the 95% confidence interval. If the upper limit of the 95% confidence interval lies below 0% (or 1 on the odds ratio scale) then superiority of the intervention will be concluded with respect to the primary safety outcome.

For the primary analysis, the data will be analysed according to the actual timing of the introduction of the new pathway rather than the randomised time the new pathway was to be introduced (if different).

Sample size:

Based on simulation methods, the total sample size of 38,994 patients will provide 99% power at the two-sided 5% level of significance to detect a realistic true difference of at least 60 minutes in arithmetic mean length of stay (primary efficacy endpoint).

Again, based on simulation methods, a sample size of 38,994 patients ensures that there is 90% power to demonstrate non-inferiority assuming an event rate of 0.4% for the primary safety end-point (type 1 or type 4b myocardial infarction or cardiac death from discharge to 30 days), a one-sided 95% confidence interval is constructed for the difference, and a non-inferiority margin of 0.5% in favor of standard care. The 0.4% event rate assumption comes from initially reported observations in the validation phase of the HighSTEACS trial.

The overall power to detect a statistically significant difference for the primary efficacy endpoint *and* demonstrate non-inferiority for the primary safety endpoint is 89%.

8. Expected Results

Based on our previous studies we expect the ARCHITECT STAT high-sensitive troponin I assay to reclassify up to 17% of all patients admitted with suspected acute coronary syndrome: a potential 46% increase in the diagnosis of myocardial infarction. We anticipate that clinical outcomes will improve in those patients reclassified as myocardial infarction due to greater use of effective therapies for the treatment of coronary artery disease. It is likely that this improvement in outcome will be at the expense of more patients identified with elevated cardiac troponin concentrations with non-cardiac causes of chest pain. The use of cardiac medication and investigations in this group is likely to increase, but may not improve clinical outcomes.

These findings will represent the first evidence from a randomised controlled trial to evaluate the appropriateness of the universal definition of myocardial infarction and to assess its impact on clinical outcomes. One of the major strengths of the trial design is that we will capture all patients with suspected acute coronary syndrome presenting to both secondary and tertiary care hospitals rather than a highly selected sub-population of consented patients managed in a regional cardiac centre. This large and unique data set will provide us with an opportunity to critically evaluate whether it is appropriate to base the diagnosis of myocardial infarction on a statistical assumption about the normal distribution of troponin from an unrelated reference population. Using this novel high sensitive assay we will quantify troponin in >98% of study participants and therefore will be able to examine the true relationship between troponin concentration and clinical outcome to define the optimal clinical threshold or parameters that identify patients who would benefit from treatment for myocardial infarction.

National guidelines now recommend the use of high-sensitivity cardiac troponin assays to rule out of myocardial infarction earlier in patients with suspected acute coronary syndrome. These assays will allow earlier testing with serial measurements performed in the Emergency Department and will permit use of lower thresholds such that myocardial infarction may be safely ruled out on presentation. This trial will determine whether the introduction of early rule out pathways into routine clinical practice is safe.

We anticipate that implementation of a novel high-sensitivity cardiac troponin assay and an early rule out pathway will be associated with major cost savings, and our findings will form the basis of future guidelines for the assessment of patients with suspected acute coronary syndrome.

9 Safety Reporting

Safety endpoints will be monitored throughout the main study and form part of routine reports. The Trial Management Team, the Trial Steering Committee (TSC) and the data monitoring committee (DMC) will oversee safety monitoring throughout the study. Individual adverse events will not be monitored as part of the main study. In the event of any safety concerns being reported to the trial team, these will be recorded and reported to the Sponsor and the Trial Steering Committee within the appropriate regulatory timeframes.

10 Study Monitoring

10.1 Trial Steering Committee

The committee will include representatives from the grant applicants, trial management and four individuals not directly involved in the trial. A member of a relevant consumer (patient) group as well as a representative of the British Heart Foundation (BHF) will be invited to be members of the committee.

10.2 Trial Management Group

The trial management group will meet regularly and consists of the grant applicants, the trial manager, and research team.

10.3 Data Monitoring Committee

The main study involves linkage of records from existing patient records and does not require data entry. A data monitoring committee will oversee the data management and record linkage processes throughout the study.

10.4 End-point adjudication committee

An endpoint adjudication committee will also be set up to adjudicate the study endpoints and validate documented endpoints.

11 Ethical and Regulatory Issues

The High-STEACS study protocol V 1.0 was approved by the Scotland A Research Committee on 14th August 2012 (Ref: 12/SS/0115). NHS Lothian Research and Development Approval (2012/R/CAR/17) has been obtained and is in progress in NHS Greater Glasgow and Clyde.

12 References

ACCF/AHA Task force, 2013 ACCF/AHA key data elements and definitions for measuring the clinical management and outcomes of patients with acute coronary syndromes and coronary artery disease. *J Am Coll Cardiol.* 2013;61(9)

Apple FS, Simpson PA, Murakami MM. Defining the serum 99th percentile in a normal reference population measured by a high-sensitivity cardiac troponin I assay. *Clin Biochem.* 2010;43(12):1034-6.

Apple FS, Collinson PO. Analytical characteristics of high-sensitivity cardiac troponin assays. *Clin Chem.* 2011 Sept 30. [Epub ahead of print]

Bauer A, Gawaz M. Sensitive cardiac troponin assays. *N Engl J Med* 2009;361:2575.

Collinson P, van Dieijen-Visser MP, Pulkki K, et al. Evidence-based laboratory medicine: how well do laboratories follow recommendations and guidelines? The Cardiac Marker Guideline Uptake in Europe (CARMAGUE) Study. *Clin Chem* 2011a. Sept 27. [Epub ahead of print]

Collinson P, Goodacre S, Gaze D, Gray A. Very early diagnosis of chest pain by point-of-care testing: comparison of the diagnostic efficiency of a panel of cardiac biomarkers compared with troponin measurement alone in the RATPAC trial. *Heart.* 2011b. Nov 10. [Epub ahead of print]

Eggers KM, Jaffe AS, Lind L, Venge P, Lindahl B. 2009. Value of cardiac troponin I cutoff concentrations below the 99th percentile for clinical decision-making. *Clin Chem* 2009;55(1):85-92.

Goodacre S, Cross E, Arnold J, Angelini K, Capewell S, Nicholl J. The health care burden of acute chest pain. *Heart* 2005;91(2):229-230.

Keller T, Zeller T, Peetz D, Tzikas S, Roth A, Czyz E, et al. Sensitive troponin I assay in early diagnosis of acute myocardial infarction. *N Engl J Med* 2009;361(9):868-877.

Keller T, Munzel T, Blankenberg S. Making it more sensitive: the new era of troponin use. *Circulation* 2011;123(13):1361-1363.

Keller, T., Zeller, T., Ojeda, F., Tzikas, S., Lillpopp, L., Sinning, C., Wild, P., et al. Serial changes in highly sensitive troponin I assay and early diagnosis of myocardial infarction. *JAMA 2011;* 306(24): 2684–2693.

Lindahl B, Venge P, James S. The new high-sensitivity cardiac troponin T assay improves risk assessment in acute coronary syndromes. *Am Heart J* 2010;160(2):224-229.

Matias M, Prostko J, Palafox M, et al. Analytical Performance of the ARCHITECT *STAT* High-sensitive Troponin I Immunoassay. *Clinical Chemistry* 2010;56(6):A121.

McFalls EO, Larsen G, Johnson GR, et al. Outcomes of hospitalized patients with non-acute coronary syndrome and elevated cardiac troponin level. *Am J Med* 2011;124(7):630-635.

Mills NL, Walker S, Newby DE. 2011. Sensitive troponin I assay in patients with suspected acute coronary syndrome. *JAMA* 2011a;306(5):489.

Mills NL, Churchhouse AM, Lee KK, et al. Implementation of a sensitive troponin I assay and risk of recurrent myocardial infarction and death in patients with suspected acute coronary syndrome. *JAMA* 2011b;305(12): 1210-1216.

Mills NL, Lee KK, McAllister DA, et al. Implications of lowering the threshold for plasma troponin in the diagnosis of myocardial infarction. *BMJ* 2011c; manuscript under revision.

Morrow DA, Cannon CP, Jesse RL, et al. National Academy of Clinical Biochemistry Laboratory Medicine Practice Guidelines: clinical characteristics and utilization of biochemical markers in acute coronary syndromes. *Clin Chem* 2007;53(4):552-574.

Reichlin T, Hochholzer W, Bassetti S, et al. Early diagnosis of myocardial infarction with sensitive cardiac troponin assays. *N Engl J Med* 2009;361(9):858-867.

Reichlin T, Irfan A, Twerenbold R, et al. Utility of absolute and relative changes in cardiac troponin concentrations in the early diagnosis of acute myocardial infarction. *Circulation.* 2011;124(2):136-45.

Sabatine MS, Morrow DA, de Lemos JA, Jarolim P, Braunwald E. Detection of acute changes in circulating troponin in the setting of transient stress test-induced myocardial ischaemia using an ultrasensitive assay: results from TIMI 35. *Eur Heart J* 2009;30(2):162-169.

Thygesen K, Alpert JS, White HD, et al. Universal definition of myocardial infarction. *Circulation* 2007;116(22):2634-2653.

Wallentin L, Becker RC, Budaj A, et al. Ticagrelor versus clopidogrel in patients with acute coronary syndromes. *N Engl J Med*. 2009;361:1045-1057.

13 Appendices

Appendix 1

*High-S*ensitive *T*roponin in the *E*valuation of patients with *A*cute *C*oronary *S*yndrome (*HighSTEACS): A* randomised controlled trial

British Heart Foundation Special Project Grant

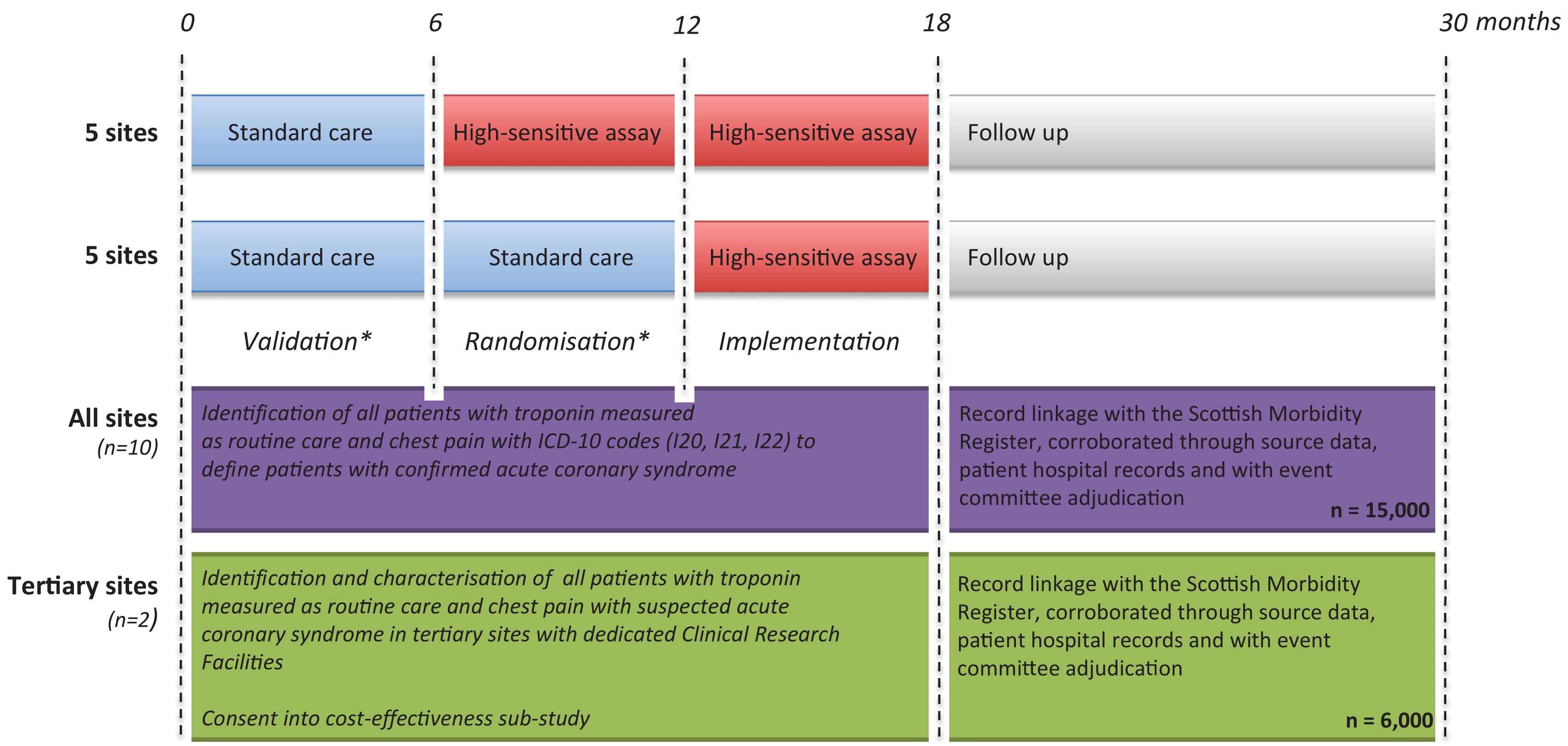

** During the validation and randomisation phases of the study both the standard and high sensitivity assays will be run in parallel. During the validation phase and following randomisation in those sites randomised to standard care, only results of the standard assay will be made available with clinical decisions based on existing diagnostic thresholds.*

Extension of implementation phase (HighSTEACS)

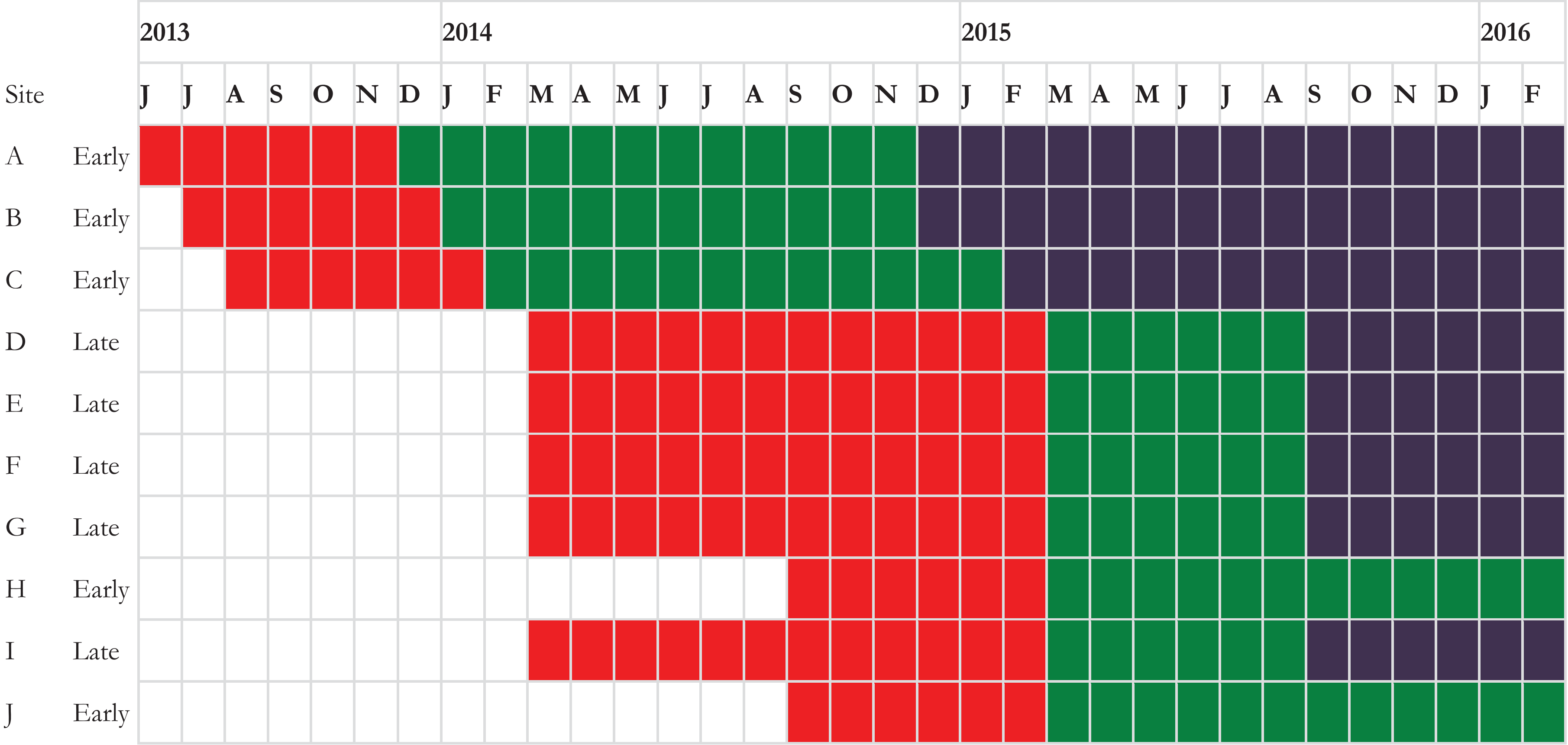

Recruitment and follow up timescales: HighSTEACS and sub studies

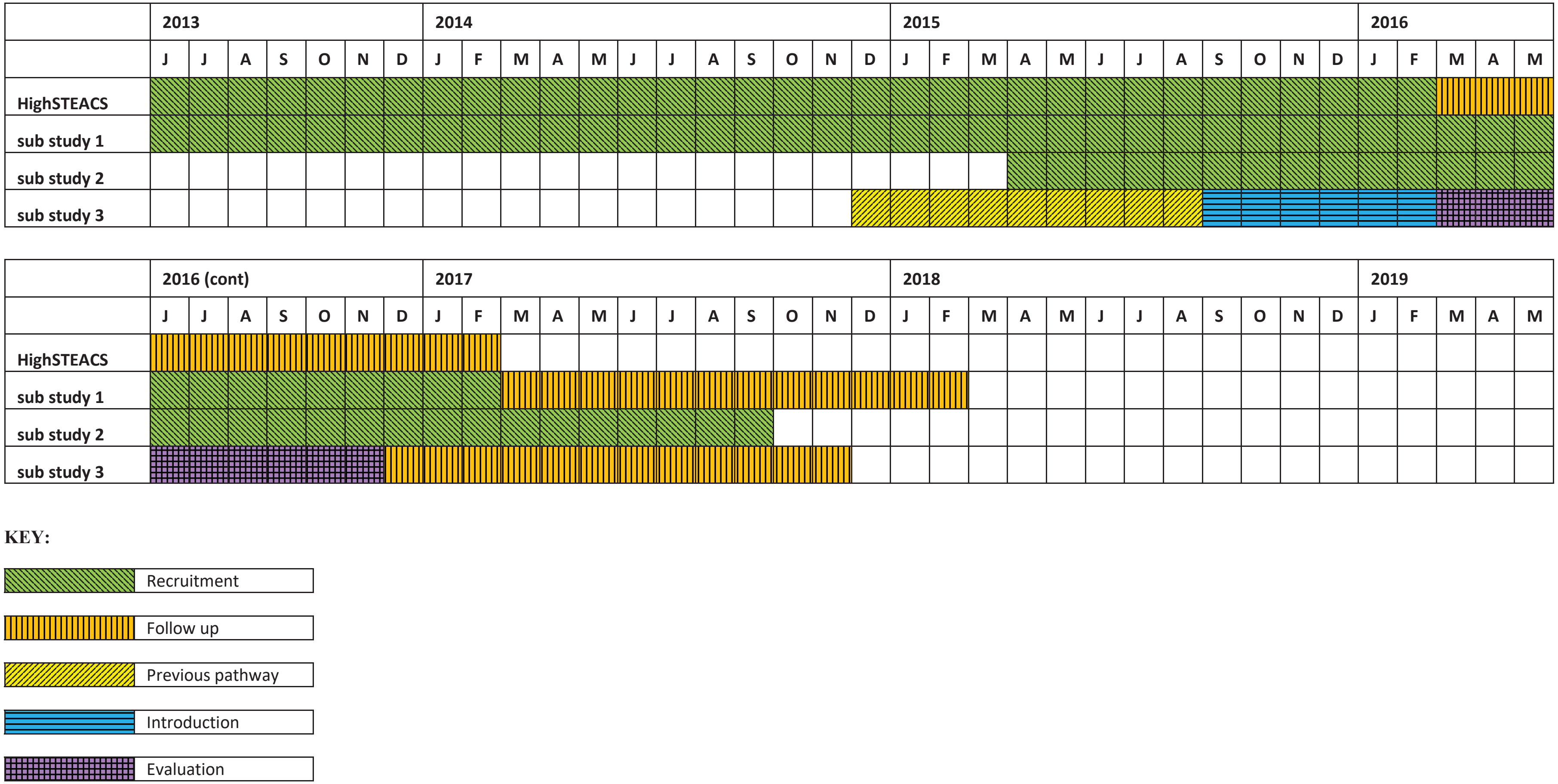

Appendix 2

Number of hospitalisations with chest pain (ICD code R7), myocardial infarction (I21, I22) and unstable angina (I20), and number of troponin assays performed on the ARCHITECT platform in all participating centres for the financial year 2008-2009.

Participating centre	Chest pain	MI	UA	TnI/annum
Vale of Leven General Hospital	455	132	72	2,548
Inverclyde Royal Hospital	980	135	175	5,486
Royal Alexandra Hospital	1,371	178	380	7,675
Glasgow Royal Infirmary	1,791	227	349	10,026
Victoria Infirmary	1,670	315	112	9,349
Southern General Hospital	1,274	163	99	7,132
Western Infirmary/ Gartnavel	1,867	187	302	10,451
Western General Hospital	379	103	115	2,121
St John's Hospital	1,001	211	286	5,604
Royal Infirmary of Edinburgh	2,051	864	588	11,482
**TOTAL**	**12,839**	**2,515**	**2,478**	**71,874**

Source: ISD Scotland, SMR01 (Inpatient/Day cases) linked database as at 26 February 2011.

MI = myocardial infarction; UA = unstable angina; TnI = troponin I

Appendix 3

Sub study 2 outline

A qualitative investigation of patients' views on and experiences of discharge in suspected acute coronary syndrome before and after implementation of early rule out strategies

Background

The HighSTEACS study is assessing the impact of the introduction of a new high-sensitivity troponin assay on the clinical management of acute coronary syndrome and on clinical outcomes. These new high-sensitivity troponin assays are able to detect lower levels of cardiac troponin and have the potential to allow rule out of myocardial infarction on presentation, rather than at 12 hours with a conventional assay. High-sensitivity assays will therefore support the introduction of early discharge protocols for patients with chest pain. In this qualitative sub-study, we will explore patients' perceptions and experiences of standard and early discharge protocols.

The majority of patients with chest pain will have normal troponin values and are informed they have "non-cardiac chest pain", or "unexplained chest pain". Patients with unexplained chest pain suffer from increased anxiety, use more hospital services, and have functional status that are comparable to those patients with coronary heart disease. Studies exploring the information needs of patients with unexplained chest pain have highlighted themes of "lack of focus on individual problems","unanswered questions", and "unmet information needs". Before adopting an early discharge protocol we need to understand the patients' beliefs, perceptions and experiences related to attending hospital with chest pain. It is these beliefs and concerns that are likely to drive repeat attendance if these are not tackled during their index admission. These studies will determine the patients' view and acceptability of an early discharge protocol. We will identify whether these views differ by age and between men and women. We anticipate that an early discharge protocol for all patients may increase patient anxiety. Our findings will help identify ways to support patients following early discharge, and lead to clinical guidance tools that will reduce the risk of inappropriate discharge or reattendance.

Research Methods: This study will utilize a mixed methods approach. Patients with suspected acute coronary syndrome in whom myocardial infarction has been ruled out will be recruited from the Emergency Department and Medical Assessment Areas of the Royal Infirmary Edinburgh, Western General Hospital Edinburgh, and St John's Hospital Livingston before (n=40; phase 1) and after (n=40; phase 2) implementation of the early discharge protocol. Patients will not be made aware that discharge may be occurring at an earlier time point than conventional treatment so as not to potentially load any anxiety. Patients will be stratified by age and gender with 10 subjects in each group (males ≤65 and >65 years; females ≤65 and >65 years based on the mean age of presentation with suspected acute coronary syndrome). Patient's co-morbid illness, social support and levels of anxiety will be recorded quantitatively using a visual analogue scale completed on discharge and at the home visit approximately 1 week later, and a Hospital Anxiety and Depression scale (HAD) validated for use in NCCP patients. Follow up investigations arranged for this group of patients, and their outcomes at 1 year will also be recorded. The qualitative component of the study will use a phenomenological approach that explores lived experience, revealing both what is experienced and how it is experienced. In- depth interviews will be conducted, with participant consent, in the week following discharge. Interviews will be recorded and transcribed and an analytical framework developed. Transcripts will be read multiple times in order to develop relevant codes and analytical themes. The interview schedule will be developed after a thorough literature review has been conducted and tested through pilot interviews. A preliminary scoping of the literature suggests four interrelated themes, but we shall remain alert to themes emerging from the patients' accounts. Based on previous studies and a literature review I will explore four themes

- Patients' beliefs of the cause of chest pain
- Patients' experiences of how a cardiac cause has been ruled out
- The interrelationship between health care seeking behaviour, self-care and social support
- Patients' experience of standard of care

Recruitment
Patients attending the Royal Infirmary of Edinburgh (Emergency Department or Primary Assessment Area), the Western General Hospital Edinburgh (Acute Receiving Unit), or St John's Hospital Livingston (Emergency Department) for assessment of suspected acute coronary syndrome will be offered the opportunity to participate in the additional qualitative study by Emergency Department clinical staff. After reading the information sheet, patients will be asked if they would be happy to be contacted by phone or e-mail at least twenty four hours later to discuss taking part in the interview study, and if so to arrange a suitable time for the interview to take place. This information will be recorded on a screening log. Those agreeing to take part in the study will then be telephoned on the day before or the morning of the interview to see if they are still happy to take part. Patient consent will be gained prior to the interview commencing.

Study procedures
Consenting patients will be visited in their own home (or place of their choosing) approximately one week after discharge. In-depth interviews surrounding their experience of discharge from hospital following an episode of chest pain will be conducted and audio recorded. Follow up investigations ordered for these patients and their outcomes at 1 year will be recorded.

Data management
All data will be gathered, stored, and transferred in a manner which is safe, ensures accuracy, maintains participant confidentiality yet remains accessible to research staff. Data will be anonymised as soon as practicable and electronic devices used for data collection will have appropriate security settings enabled to prevent unauthorised access. Any file transfers will be conducted using encryption technology (for example for professional transcription). Identifying participant details will be held separately in a secure filing cabinet. NVivo will be used for data curation and analysis.

Transcription will be conducted by a reputable organisation used by the University of Edinburgh which adheres to strict guidance, including confidentiality. Instructions will be given so that the data can be anonymised during transcription. Each transcript will have a unique identifier and a version number before being uploaded to NVivo.

All electronic data collected for the study will be stored in a designated shared drive. The data folder will only be accessible to the research team. Data stored on external media (e.g. audio digital recorders, pen drives) will be transferred to the relevant shared folder as soon as practicable. After 5 years the audio recordings will be destroyed.

All hard copy data will be stored in a locked filing cabinet in a secure office. Under no circumstances will paper records be left unattended in a public area with anyone outside the research team.

Summary: Our findings will help identify ways to support patients following early discharge, and lead to clinical guidance tools that will reduce the risk of inappropriate discharge or reattendance.

Appendix 4. Early rule out pathway for patients with suspected acute coronary syndrome (NHS Lothian)

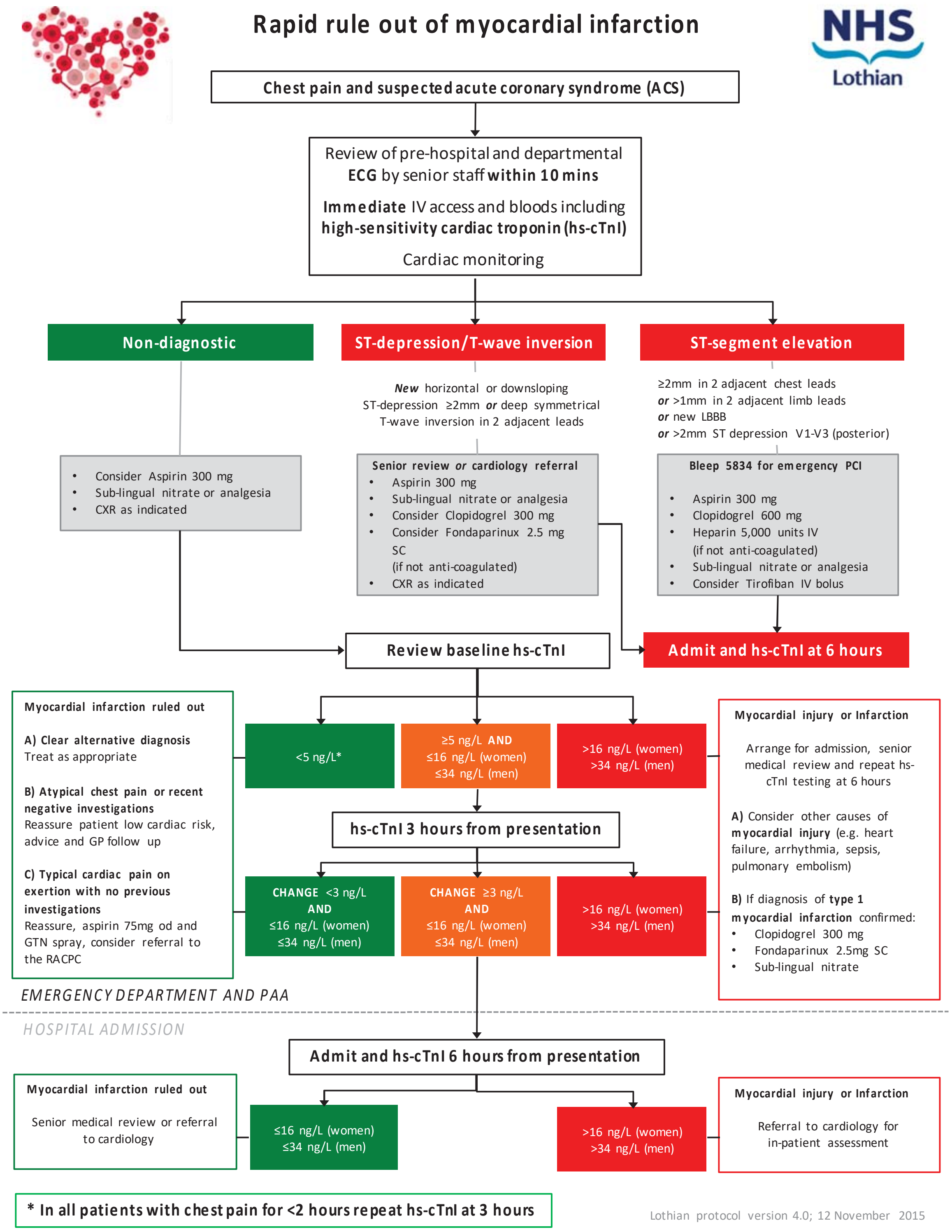

SUSPECTED ACUTE CARDIAC CHEST PAIN PROTOCOL

NHS Greater Glasgow and Clyde

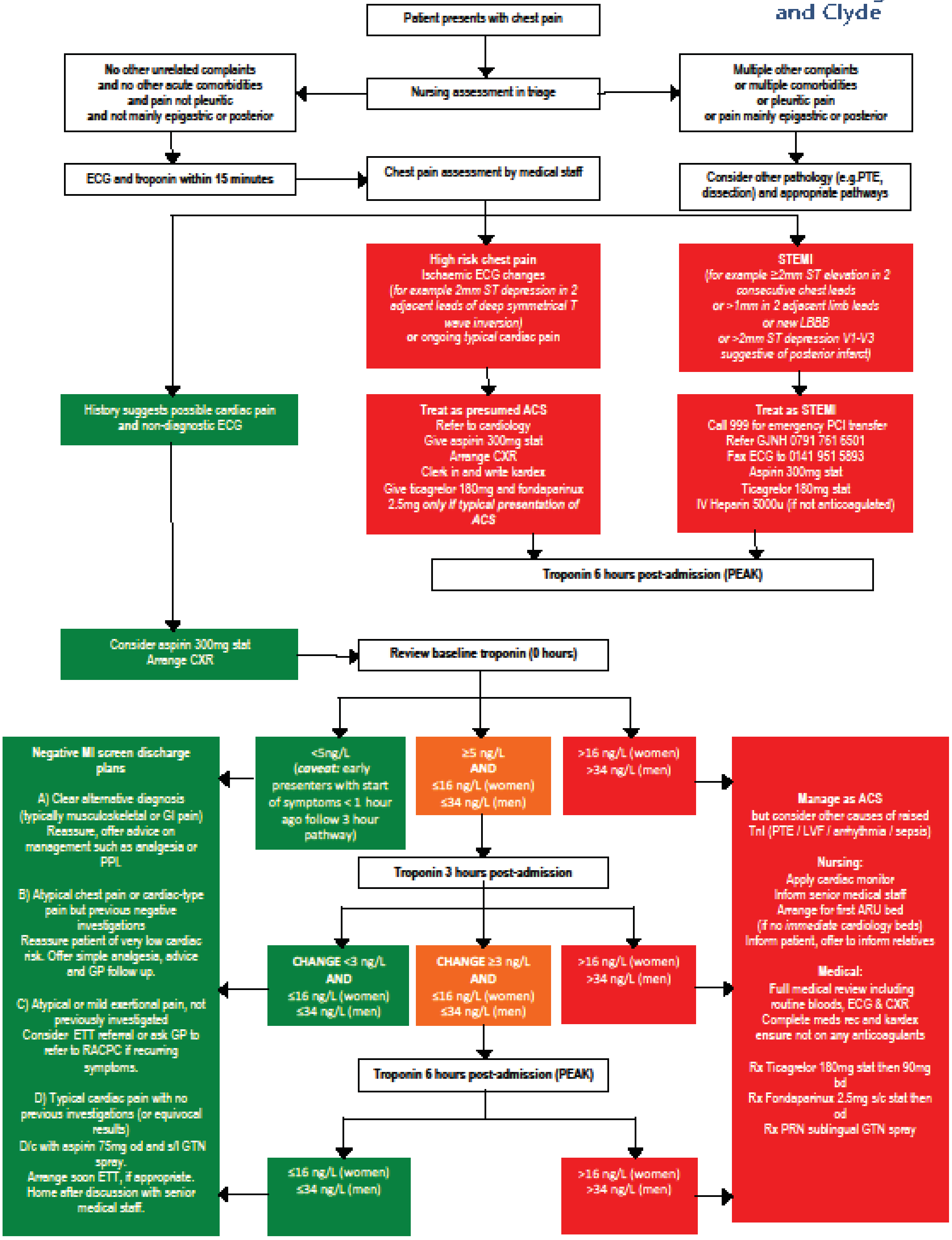

Supplement: Supplement 1. — Trial protocol [file jamainternmed-e211184-s001.pdf]
