# Supplementary material for: Use of High-Sensitivity Cardiac Troponin in Patients With Kidney Impairment: A Randomized Clinical Trial
Source: JAMA Intern Med. 2021 Jun 7;181(9):1237–9. doi: 10.1001/jamainternmed.2021.1184 (PMC8185626; doi:10.1001/jamainternmed.2021.1184)
Supplement: Supplement 2. — eAppendix. Implementation of high-sensitivity cardiac troponin in patients with renal impairment [file jamainternmed-e211184-s002.pdf]

Supplemental Online Content

Gallacher PJ, Miller-Hodges E, Shah ASV, Anand A, Dhaun N, Mills NL; High-STEACS Investigators. Use of high-sensitivity cardiac troponin in patients with kidney impairment: a randomized clinical trial. *JAMA Intern Med*. Published online June 7, 2021. doi:10.1001/jamainternmed.2021.1184

eAppendix. Implementation of high-sensitivity cardiac troponin in patients with renal impairment

This supplemental material has been provided by the authors to give readers additional information about their work.

© 2021 Gallacher PJ et al. *JAMA Internal Medicine.*

E.APPENDIX

Implementation of High-Sensitivity Cardiac Troponin in Patients with Renal Impairment

Peter J. Gallacher, MD,[1] Eve Miller-Hodges, MD,[1,2] Anoop S.V. Shah, MD,[1,3]
Atul Anand, MD,[1] Neeraj Dhaun, MD,[1,2*] Nicholas L. Mills, MD[1,4*]
on behalf of the *High-STEACS* investigators

[1] BHF Centre for Cardiovascular Science, University of Edinburgh, Edinburgh, UK.
[2] Department of Renal Medicine, Royal Infirmary of Edinburgh, Edinburgh, UK.
[3] London School of Hygiene and Tropical Medicine, London, UK
[4] Usher Institute, University of Edinburgh, Edinburgh, UK.

*Both authors contributed equally

Corresponding author:
Professor Nicholas L Mills
BHF Centre for Cardiovascular Science
University of Edinburgh
Edinburgh EH16 4SA
United Kingdom

Telephone: +44 131 242 6515
Fax: +44 131 242 6379
Email: nick.mills@ed.ac.uk

Supplementary Figures: 1

© 2021 Gallacher PJ et al. *JAMA Internal Medicine.*

Supplementary Methods

A full description of the trial design has been published previously,[1] but an overview is provided in the text below.

Randomization

Block randomization was used with sites paired based on the expected number of presentations and one site randomised to early implementation and the other to late implementation. The randomization sequence was generated by a programmer at the Edinburgh Clinical Trials Unit who was not otherwise involved in the study using computer generated pseudo-random numbers.

Intervention

Consecutive patients with suspected acute coronary syndrome underwent cardiac troponin testing at presentation and again 6 to 12 hours after the onset of symptoms, at the discretion of the attending clinician consistent with national[2] and international[3] guidelines. During both phases of the trial, all patients underwent testing with contemporary cardiac troponin I (ARCHITECT*STAT* troponin I, Abbott Laboratories, Abbott Park, IL, USA) and high-sensitivity cardiac troponin I (ARCHITECT*STAT* high-sensitive troponin I, Abbott Laboratories, Abbott Park, IL, USA) assays. During the pre-implementation phase of the trial, results of the high-sensitivity assay were suppressed from attending clinicians; during the implementation phase of the trial, results of the contemporary assay were suppressed. For the contemporary assay, a single threshold for the diagnosis of myocardial infarction in women and men was used to guide clinical decisions during the pre-implementation phase of the trial. This threshold was based on the local evaluation of the lowest concentration where the intra-assay coefficient of variation was <10%, which was 40 ng/L at seven sites and 50 ng/L at three sites. For the high-sensitivity

© 2021 Gallacher PJ et al. *JAMA Internal Medicine.*

assay, the coefficient of variation was <10% at 4.7 ng/L, and the diagnostic threshold was based on sex-specific 99th centile upper reference limits of 16 ng/L for women and 34 ng/L for men.[4]

Adjudication of the diagnosis of myocardial infarction

Type 1 myocardial infarction was defined as myocardial necrosis (any hs-cTnI concentration above the 99th centile with a rise and/or fall in hs-cTnI concentration where serial testing was performed) in the context of a presentation with suspected acute coronary syndrome with symptoms or signs of myocardial ischemia on the electrocardiogram. Type 4b myocardial infarction was defined as myocardial injury with symptoms or signs of myocardial ischemia secondary to stent thrombosis demonstrated on coronary angiography.

Outcomes

All trial data were collected from routine electronic regional and national healthcare datasets, linked, anonymized and held securely within the NHS National Services Scotland national safe haven. All in-hospital and community deaths, and all hospital admissions are recorded on the Register of Deaths in Scotland and the Scottish Morbidity Record (SMR) respectively.

All attendances across any participating hospital where cardiac troponin was measured and the high-sensitivity cardiac troponin I concentration was above the 99th centile were reviewed and the diagnosis adjudicated. We used the same approach to adjudication as for the index hospital episode with the panel blinded to all cardiac troponin measurements during the index episode and to the study phase.

The primary outcome was myocardial infarction (type 1 or type 4b) or cardiovascular death at 1 year. Secondary efficacy end-points include myocardial infarction, unplanned coronary

© 2021 Gallacher PJ et al. *JAMA Internal Medicine.*

revascularization, cardiovascular death, all-cause death, duration of stay, hospitalization for heart failure and ischemic stroke. Secondary safety end-points include major hemorrhage, unplanned hospitalization excluding acute coronary syndrome, and non-cardiovascular death.

Unplanned coronary revascularization was defined as any urgent or emergency percutaneous coronary intervention or coronary artery bypass grafting following discharge. International Classification of Disease (ICD)-10 codes from the Scottish Morbidity Record were used to define hospitalization for heart failure (I50) and ischemic stroke (I63, I65, or I66). Bleeding was defined according to the Bleeding Academic Research Consortium (BARC) definition using ICD-10 and OPCS codes to classify each bleeding event as previously described.[(5,6)] Major hemorrhage was defined as BARC type 3 or type 5. Unplanned hospitalization excluding acute coronary syndrome was defined as any hospital attendance or admission excluding type 1 or type 4b myocardial infarction at 30 days.

Statistical analysis

A total of 48,282 consecutive patients with suspected acute coronary syndrome were enrolled across ten hospitals (**Supplementary figure 1**). Serum creatinine concentrations were available in 46,927 (97%) patients (61±17 years; 53% [n=24,980] men; 47% [n=21,947] women; 19% [n=9,080] renal impairment). The primary outcome was compared before and after implementation of the high-sensitivity assay in patients with high-sensitivity cardiac troponin concentrations above the 99th centile, stratified by eGFR (<60 and ≥60 ml/min/1.73m^2), using two identical Cox regression models. This model included all variables from the primary analysis of the High-STEACS trial.[(1)] However, as patients with renal impairment were significantly older and had more comorbidities than patients with normal renal function, we included additional adjustment for comorbidities, in accordance with the STROBE

© 2021 Gallacher PJ et al. *JAMA Internal Medicine.*

guidelines.[7] The Cox models for the stratified analysis adjusted for age, sex, study phase, hospital site (fitted as a random effect), seasonality, time of presentation from the start date of the trial, prior diabetes mellitus, ischemic heart disease or cerebrovascular disease, high-sensitivity cardiac troponin I concentration (log-transformed) and social deprivation status (SIMD quintile). All statistical analyses were performed using R, version 3.6.1 (R Foundation, Vienna, Austria).

Ethical approval and statement of data transparency

The trial was conducted in accordance with the Declaration of Helsinki and with the approval of the Scotland Research Ethics Committee, the Public Benefit and Privacy Panel for Health and Social Care and each National Health Service Health Board. As randomization was at the hospital level, individual patient consent was not sought. The High-STEACS trial makes use of multiple routine electronic health care data sources that are linked, de-identified and held in our national safe haven, which is accessible by approved members of the research team who have undertaken the necessary governance training. Summary data and source analysis code can be made available upon request to the corresponding author (nick.mills@ed.ac.uk).

© 2021 Gallacher PJ et al. *JAMA Internal Medicine.*

The High-STEACS Investigators

Chief Investigator: Professor Nicholas L Mills.

Trial managers: Dr Fiona E Strachan and Mr Christopher Tuck.

Trial research team: Dr Anoop SV Shah, Dr Atul Anand, Dr Tariq Farrah, Dr. Nynke Halbesma, Mr James Blackmur, Dr Andrew R Chapman, Dr. Fiona E Strachan, Dr Amy V Ferry, Dr Kuan Ken Lee, Mr Dennis Sandeman, Dr Philip D Adamson, Dr Catherine L Stables, Dr Catalina A Vallejo, Dr Athanasios Tsanasis, Ms Lucy Marshall, Ms Stacey D Stewart, Dr Takeshi Fujisawa, Ms Mischa Hautvast, Ms Jean McPherson and Ms Lynn McKinlay.

Grant applicants: Prof Nicholas L Mills (Principal Applicant), Prof David E Newby, Prof Keith AA Fox, Prof Colin Berry, Dr Simon Walker, and Prof Christopher J Weir.

Trial steering committee: Prof Ian Ford (chair, independent), Prof Nicholas L Mills, Prof David E Newby, Prof Alasdair Gray, Prof Keith AA Fox, Prof Colin Berry, Dr Simon Walker, Prof Paul O Collinson, Prof Fred S Apple, Mr Alan Reid, Dr Anne Cruikshank, Dr Iain Findlay, Dr Shannon Amoils (independent), Dr David A McAllister, Dr Donogh Maguire, Ms Jennifer Stevens (independent), Prof John Norrie (independent), and Prof Christopher J Weir.

Adjudication panel: Dr Anoop SV Shah, Dr Atul Anand, Dr Andrew R Chapman, Dr Kuan Ken Lee, Dr Jack PM Andrews, Dr Philip D Adamson, Dr Alastair Moss, Dr Mohamed S Anwar, Dr John Hung, Prof Nicholas L Mills.

Biochemistry sub-group committee: Dr Simon Walker, Dr Jonathan Malo, Mr Alan Reid, Dr Anne Cruikshank, Prof Paul O Collinson.

Data monitoring committee: Prof Colin M Fischbacher, Dr Bernard L Croal, Prof Stephen J Leslie.

Edinburgh Clinical Trials Unit: Mrs Catriona Keerie, Mr Richard A Parker, Mr Allan Walker, Mr Ronnie Harkess, Mr Christopher Tuck, Mr Tony Wackett, Prof Christopher Weir.

© 2021 Gallacher PJ et al. *JAMA Internal Medicine.*

NHS Greater Glasgow & Clyde Safe Haven: Dr Roma Armstrong, Ms Marion Flood, Ms Laura Stirling, Ms Claire MacDonald, Mr Imran Sadat, Mr Frank Finlay.

NHS Lothian Research Governance, eHealth and Safe Haven: Dr Heather Charles, Ms Pamela Linksted, Mr Stephen Young, Mr Bill Alexander, Mr Chris Duncan.

© 2021 Gallacher PJ et al. *JAMA Internal Medicine.*

Supplementary figures

Supplementary figure 1. CONSORT diagram of original trial[9] and patient populations. Between June 10, 2013, and March 3, 2016, 48,282 consecutive patients with suspected acute coronary syndrome met the trial inclusion criteria and were included in the analysis of the primary outcome. The trial concluded on March 3, 2017, after a minimum follow-up period of 1 year.

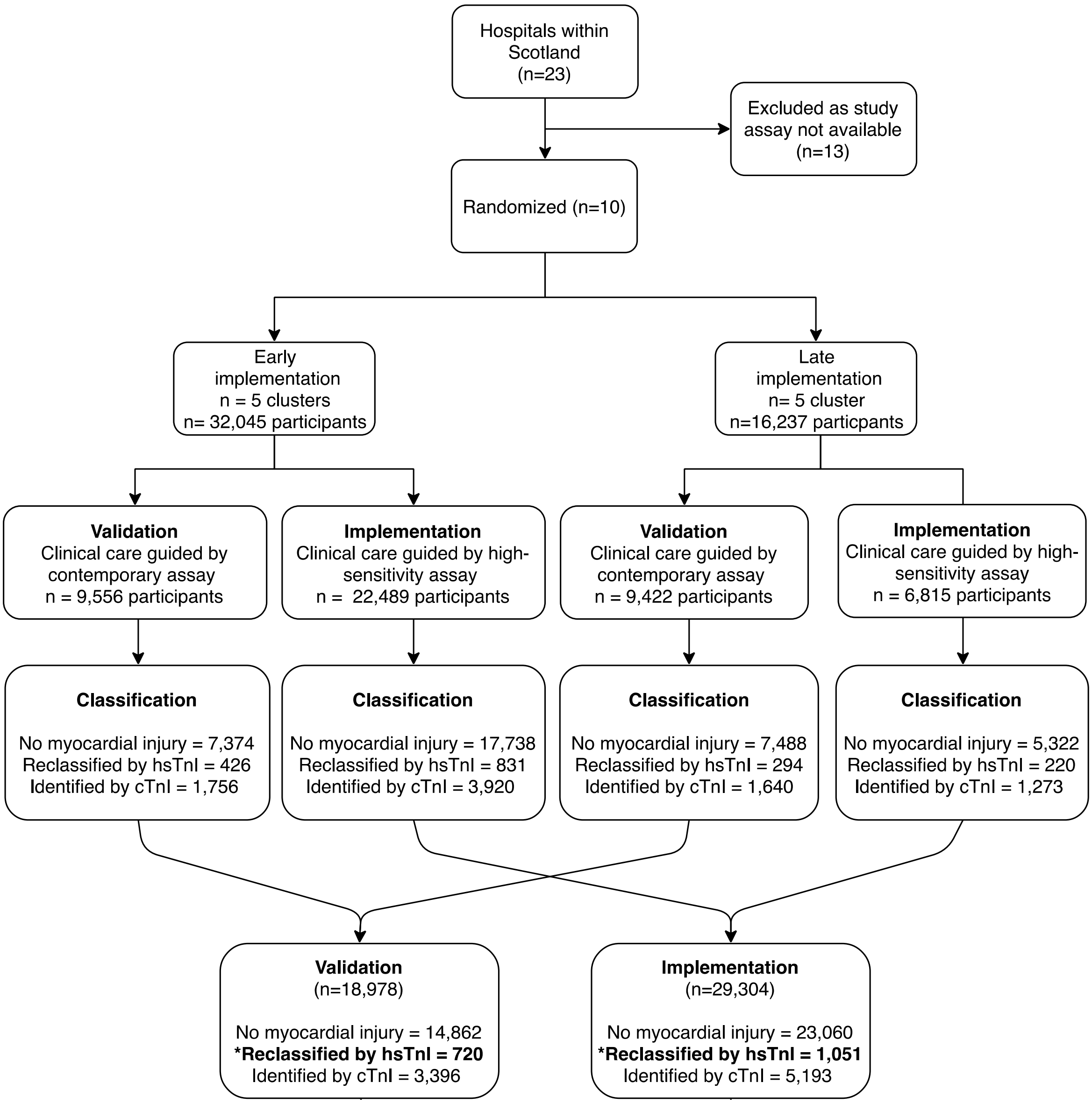

© 2021 Gallacher PJ et al. *JAMA Internal Medicine.*

References

1. Shah ASV, Anand A, Strachan FE, et al. High-sensitivity troponin in the evaluation of patients with suspected acute coronary syndrome: a stepped-wedge, cluster-randomised controlled trial. *Lancet*. 2018;392(10151):919-928.
2. Acute coronary syndromes. In: (SIGN) *SIGN*, editor. Edinburgh, 2013.
3. Roffi M, Patrono C, Collet JP, et al. 2015 ESC Guidelines for the management of acute coronary syndromes in patients presenting without persistent ST-segment elevation: Task Force for the Management of Acute Coronary Syndromes in Patients Presenting without Persistent ST-Segment Elevation of the European Society of Cardiology (ESC). *Eur Heart J*. 2016;pp. 267-315.
4. Shah AS, Griffiths M, Lee KK, et al, 2015. High sensitivity cardiac troponin and the under-diagnosis of myocardial infarction in women: prospective cohort study. *BMJ*, 350:g7873.
5. Mehran R, Rao SV, Bhatt DL, et al. Standardized bleeding definitions for cardiovascular clinical trials: a consensus report from the Bleeding Academic Research Consortium. Consensus Development Conference presented at *Circulation*; Jun 14, 2011.
6. Denaxas SC, George J, Herrett E, et al. Data resource profile: cardiovascular disease research using linked bespoke studies and electronic health records (CALIBER). *Int J Epidemiol*. 2012;41(6):1625-1638.
7. von Elm E, Altman DG, Egger M, Pocock SJ, Gøtzsche PC, Vandenbroucke JP. The Strengthening the Reporting of Observational Studies in Epidemiology (STROBE) statement: guidelines for reporting observational studies. *Lancet*. 2007;370(9596):1453-1457.

© 2021 Gallacher PJ et al. *JAMA Internal Medicine.*
