# Supplementary material for: Use of High-Sensitivity Cardiac Troponin in Patients With Kidney Impairment: A Randomized Clinical Trial
Source: JAMA Intern Med. 2021 Jun 7;181(9):1237–9. doi: 10.1001/jamainternmed.2021.1184 (PMC8185626; doi:10.1001/jamainternmed.2021.1184)
Supplement: Supplement 3. — Nonauthor collaborators. The High-STEACS Investigators [file jamainternmed-e211184-s003.pdf]

Supplemental Online Content: Nonauthor Collaborators

*Indicates required information. Only first name, last name, and suffix will appear in PubMed.

| *Group Name(s): The High-STEACS Investigators | | | | | | | |
|---|---|---|---|---|---|---|---|
| ***First Name and Middle Initial(s)** | ***Last Name** | ***Suffix (eg, Jr, III)** | Academic Degrees | Institution | Location (city, state/province, country) | Role or Contribution, eg, chair, principal investigator | Group (if more than 1 Group listed in the byline) and/or Subgroup (eg, Steering Committee) |
| Nicholas L | Mills | | | | | | |
| Fiona E | Strachan | | | | | | |
| Christopher | Tuck | | | | | | |
| Anoop SV | Shah | | | | | | |
| Atul | Anand | | | | | | |
| Tariq | Farrar | | | | | | |
| Nynke | Halbesma | | | | | | |
| James | Blackmur | | | | | | |
| Andrew R | Chapman | | | | | | |
| Amy V | Ferry | | | | | | |
| Kuan Ken | Lee | | | | | | |
| Dennis | Sandeman | | | | | | |
| Philip D | Adamson | | | | | | |
| Catherine L | Stables | | | | | | |
| Catalina A | Vallejos | | | | | | |
| Athanasios | Tsanas | | | | | | |
| Lucy | Marshall | | | | | | |
| Stacey D | Stewart | | | | | | |
| Takeshi | Fujisawa | | | | | | |
| Mischa | Hautvast | | | | | | |
| Jean | McPherson | | | | | | |
| Lynn | McKinlay | | | | | | |
| David E | Newby | | | | | | |
| Keith AA | Fox | | | | | | |
| Colin | Berry | | | | | | |
| Simon | Walker | | | | | | |
| Christopher J | Weir | | | | | | |
| Alasdair | Gray | | | | | | |
| Paul O | Collinson | | | | | | |

Supplemental Online Content: Nonauthor Collaborators

*Indicates required information. Only first name, last name, and suffix will appear in PubMed.

| *First Name and Middle Initial(s) | *Last Name | *Suffix (eg, Jr, III) | Academic Degrees | Institution | Location (city, state/province, country) | Role or Contribution, eg, chair, principal investigator | Group (if more than 1 Group listed in the byline) and/or Subgroup (eg, Steering Committee) |
|---|---|---|---|---|---|---|---|
| Fred S | Apple | | | | | | |
| Alan | Reid | | | | | | |
| Anne | Cruikshank | | | | | | |
| Iain | Findlay | | | | | | |
| David A | McAllister | | | | | | |
| Donogh | Maguire | | | | | | |
| Jack PM | Andrews | | | | | | |
| Alastair | Moss | | | | | | |
| Mohamed S | Anwar | | | | | | |
| John | Hung | | | | | | |
| Jonathan | Malo | | | | | | |
| Colin | Fischbacher | | | | | | |
| Bernard L | Croal | | | | | | |
| Stephen J | Leslie | | | | | | |
| Catriona | Keerie | | | | | | |
| Richard A | Parker | | | | | | |
| Allan | Walker | | | | | | |
| Ronnie | Harkess | | | | | | |
| Tony | Wackett | | | | | | |
| Roma | Armstrong | | | | | | |
| Laura | Stirling | | | | | | |
| Claire | MacDonald | | | | | | |
| Imran | Sadat | | | | | | |
| Frank | Finlay | | | | | | |
| Heather | Charles | | | | | | |
| Pamela | Linksted | | | | | | |
| Stephen | Young | | | | | | |
| Bill | Alexander | | | | | | |
| Chris | Duncan | | | | | | |
