# Supplementary material for: Use of High-Sensitivity Cardiac Troponin in Patients With Kidney Impairment: A Randomized Clinical Trial
Source: JAMA Intern Med. 2021 Jun 7;181(9):1237–9. doi: 10.1001/jamainternmed.2021.1184 (PMC8185626; doi:10.1001/jamainternmed.2021.1184)
Supplement: Supplement 4. — Data sharing statement [file jamainternmed-e211184-s004.pdf]

Data Sharing Statement

Gallacher. Use of High-Sensitivity Cardiac Troponin in Patients With Kidney Impairment. *JAMA Intern Med*. Published June 07, 2021. doi:10.1001/jamainternmed.2021.1184

Data
Data available: No

Additional Information
Explanation for why data not available: Access to trial data was restricted to approved members of the research team who had completed information governance training. Source analysis code can be made available upon request to the corresponding author.
